# Genotyping tool for salmonid gill pox virus (SGPV) obtained from farmed and wild Atlantic salmon (*Salmo salar*)

**DOI:** 10.1007/s00705-023-05866-8

**Published:** 2023-09-08

**Authors:** Are Nylund, Thomas Kloster-Jensen, Faezeh Mohammadi, Erwan Lagadec, Heidrun Plarre

**Affiliations:** Fish Diseases Research Group, Department of Biological Sciences, University of Bergen, Bergen, Norway

## Abstract

Poxviruses are common viruses found in vertebrate species. In 2006, the first poxvirus associated with salmon, salmonid gill poxvirus (SGPV), was identified during an outbreak of gill disease at a smolt production site in northern Norway and at two marine farms in western Norway. Poxviruses had previously been detected in ayu (*Plecoglossus altivelis*) and koi carp (*Cyprinus carpio*). In all three fish species, poxviruses are associated with gill disease. It has not been possible to culture SGPV from Norway, and little is known about its virulence. However, the association between SGPV and gill disease in salmon has shown the need for molecular tools to identify reservoirs and transmission routes. Sequencing the genome of a second isolate of SGPV has made it possible to compare variable regions between two strains of the virus, showing the presence of a large number of variable regions that exhibit both variable numbers of tandem repeats and intra-ORF variation. We present eight regions that are suitable for distinguishing strains of SGPV and determining their phylogenetic relationship, and these were used to compare SGPV isolates obtained from both farmed and wild salmon in fresh and sea water. The prevalence of the virus was found to be higher in wild salmon in rivers than in returning wild salmon collected from traps in Norwegian fjords. Genotyping based on the eight selected variable regions, suggests the presence of geographically distinct isolates in freshwater among both farmed and wild salmon, while SGPV from marine farms shows high local diversity and a wide geographical distribution of similar strains of the virus.

**Supplementary Information:**

The online version contains supplementary material available at 10.1007/s00705-023-05866-8.

## Introduction

Members of the family *Poxviridae* are grouped into two distinct subfamilies, *Entomopoxvirinae* and *Chordopoxvirinae*, with viruses infecting vertebrates belonging to the latter [1]. Poxviruses are among the earliest known viral pathogens to cause infections in mammals, and during the last two decades, members of the family *Poxviridae* have emerged as a threat to fish farming [2–18]. The first outbreak of disease associated with a poxvirus infecting salmon (*Salmo salar*) was observed in freshwater at a smolt production site in northern Norway in November 2005 [4–6]. The fish were lethargic, stopped eating, and showed clear signs of respiratory problems. The total mortality at the site reached about 20%. Examination by transmission electron microscopy showed that the gill disease was associated with a poxvirus, which was named “salmonid gill poxvirus” (SGPV) [6]. In 2006, the same virus was associated with gill disease at a marine production site in western Norway, where the mortality rate was close to 90% [5, 6]. A range of different pathogens were present on the gills of the salmon at this site [4–6], and the official diagnosis was amoebic gill disease associated with a new species of *Paramoeba* later described as *Paramoeba perurans* [19]. A partial genome sequence of the SGPV isolate and phylogenetic analysis showing its position in a putative early branch of the subfamily *Chordopoxvirinae* were published in 2015 [10]. Implementation of real-time RT PCR targeting the genome of SGPV has shown that the virus is frequently present at salmon smolt production sites and marine production sites in all parts of Norway, often in association with gill disease [7, 11–13, 20, 21]. The virus, which seems to be specific for salmon, is also present in wild salmon [22]. Though the natural reservoir seems to be limited to wild and farmed salmon, much work is needed to establish the transmission routes for SGPV, differences in virulence among strains, and the molecular basis for virulence.

Poxvirus genomes range in size from 130 to 456 kbp, encoding 150 to 392 proteins, depending on the species [1, 23]. Carp edema virus (CEV) has the largest genome, (456 kbp) while the genome of SGPV is about 242 kbp in length [10, 23]. The most conserved proteins, which are essential for virus transcription, replication, and virion assembly, are encoded in the central region of the poxvirus genome, while the less-conserved proteins, which are involved in host specificity and virulence, are encoded in the terminal regions [1, 24]. Hence, it is expected that sequences from the terminal regions of the SGPV genome should be the most suitable for identifying intraspecies variation. The ends of poxvirus genomes contain inverted terminal repeats and duplicated genes. They also contain stretches of variable numbers of tandem repeats (VNTRs), and a multi-locus VNTR analysis has already been carried out for several SGPV isolates collected from Atlantic salmon [25]. However, in studies of reservoirs and transmission of poxvirus, sequences of individual genes (ORFs) or whole genomes are typically used to determine phylogenetic relationships [26–31]. In this study, comparisons were based on eight genome fragments (ORF and intergenic sequences) ranging in size from 487 to 1340 nucleotides.

It has not been possible so far to culture SGPV from salmon in the eastern part of the North Atlantic. To locate variable regions in the genome of SGPV, a partial genome sequence of SGPV was determined by Illumina sequencing of positive gill tissue from salmon collected in Hordaland County. This partial genome sequence was compared with that of an SGPV isolate from Nordland County (accession no. KT159937), the latter of which was published by Gjessing et al. [10]. Eight variable regions were selected to determine the relationships between a selection of SGPV isolates from farmed and wild salmon in fresh and sea water in Norway. The present study presents the first multi-locus sequence analysis (MLSA) for distinguishing different strains of SGPV based on phylogenetic relationships.

## Materials and methods

### Collection of fish tissues and SGPV

Gill tissue samples from farmed Atlantic salmon (*Salmo salar*) were collected from several locations in fresh- and seawater on the coast of Norway. The salmon were made available by different companies, and the tissues were sampled on site or at the Fish Diseases Research Group (FDRG) laboratory at the University of Bergen. Fish samples originating from companies were collected from licensed Norwegian fish farms (Norwegian Directorate of Fisheries). The fish were treated by aqua-medicine biologists according to the Norwegian Welfare Act (01.01.2010), and the study strictly followed the regulations set by the Norwegian Food Safety Authority. The tissues were stored at -40 °C.

**Fig. 1:**
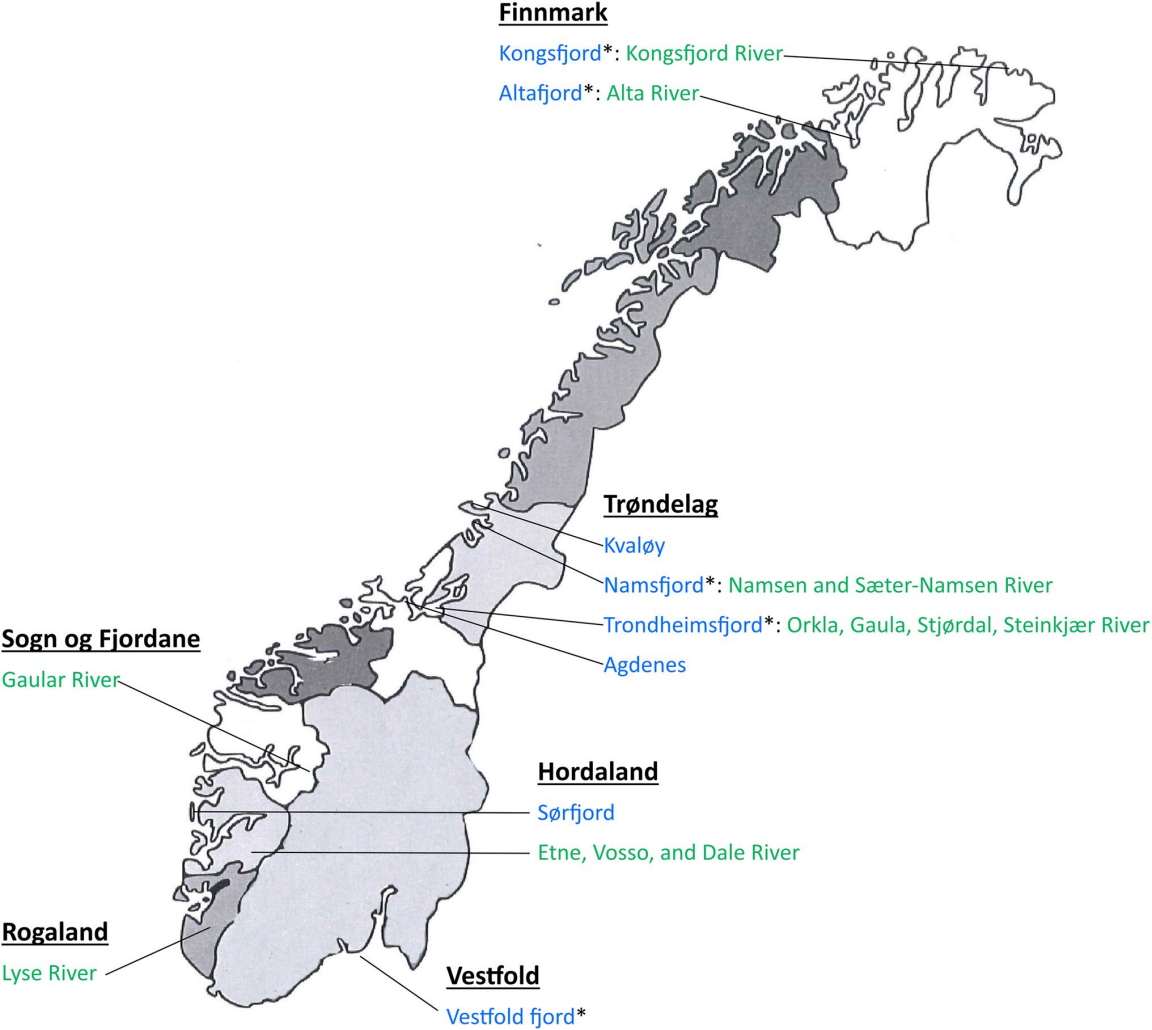
Location of the sites where wild salmon were collected. Administrative names of Norwegian counties are shown in bold and underlined; seawater collection sites are indicated in blue; rivers are indicated in green. The symbol “*” indicates fjords with the status of protected National Salmon Fjord. Wild salmon in the sea were collected using fish traps (kilenot), and salmon in rivers were collected by fly fishing.

Wild Atlantic salmon and trout (*Salmo trutta*) from rivers in western Norway (Hordaland and Sogn og Fjordane), central Norway (Trøndelag), and northern Norway (Finnmark) were collected by fly-fishing during the sportfishing season. Salmon broodfish from the Skjern River (Denmark) and the Etne, Vosso, and Dale rivers (Norway) were kept in tanks together before sampling. Wild salmon and trout in the sea (Vestfold, Sørfjorden, Agdenes, Kvaløy, Namsfjorden, Altafjorden, and Kongsfjorden) were collected in fish traps (Kilenot). All fish traps were in national salmon fjords, i.e., fjords without salmon farming, except the fish trap located at Kvaløy. However, high densities of salmon farms are present on the coastline surrounding the fjord mouths, which means that salmon returning to these national salmon fjords are likely to be exposed to high infection pressure from different pathogens from farmed salmon. The fish traps located in Vestfold represent an exception, since there is no salmon farming in this part of Norway. An overview of the counties from where the wild salmon and trout were collected is given in Figure 1. The tissues from the wild salmon were fixed on site in 70% ethanol and transported to the FDRG laboratory. The ethanol-fixed tissues were stored at -40 °C after arrival.

The gill tissues from trout and farmed and wild salmon were used for RNA and DNA extraction.

### RNA and DNA extraction

RNA was extracted from individual tissue samples as described by Steigen et al. [32]. The RNA was used for Illumina sequencing, RT-PCR, Sanger sequencing, and real-time RT-PCR. The last of these methods was used for detection of the SGPV genome in trout and farmed and wild salmon in fresh and sea water.

DNA was extracted from 30 mg of gill tissues using an E.Z.N.A. Tissue DNA kit (Omega Bio-tek) as recommended by the manufacturer. The concentration and purity of extracted DNA was evaluated using a NanoDrop ND-1000 spectrophotometer (NanoDrop Technologies). Nucleic acid samples were stored at -25 °C and used for sequencing of the selected variable regions.

### Illumina sequencing

Illumina sequencing was performed by BaseClear (BaseClear Group, The Netherlands) with an established pipeline for separation of viral RNA from salmon RNA. The RNA was obtained in May 2015 from gill tissue (Ct value = 19.6) of a positive Atlantic salmon smolt in western Norway. As poxvirus genomes contain open reading frames (ORFs) on both strands, we expected that one ORF would cover the gap between two ORFs on the complementary strand. Sequencing libraries were created using a TruSeq RNA Library Preparation Kit (Illumina). Paired-end sequence reads were generated using an Illumina HiSeq2500 system. FASTQ sequence files were generated using the Illumina Casava pipeline version 1.8.3. Initial quality assessment was based on data passing the Illumina Chastity filtering. Subsequently, reads containing adapters and/or a PhiX control signal were removed. A second quality assessment was based on the remaining reads using the FASTQC quality control tool version 0.10.0. Analysis of the results was performed using the “*de novo* assembly” option of CLC Genomics Workbench version 8.0. Misassemblies and nucleotide disagreement between the Illumina data and the contig sequences were corrected using Pilon [33] version 1.11. BLAST searches against the NCBI database were performed using the scaffold sequences generated by *de novo* assembly [34]. The BLAST results were processed using Baseclear to obtain the BLAST taxonomy ID and the corresponding taxonomic lineage. Based on the taxonomic lineage of each scaffold, the scaffold sequences were divided into the categories vertebrates, bacteria, viruses, eukaryotes (excluding vertebrates), and no match with the above-mentioned categories.

Virus scaffolds were identified using BLAST search, and a large number were identified as possible poxvirus sequences. These sequences were mapped to a previously published partial genome sequence of SGPV ([10], accession no. KT159937), and gaps were closed by Sanger sequencing. To confirm areas of sequence variation between the two SGPV genomes, we designed primers flanking each variable sequence and performed Sanger sequencing in both directions. The genome sequence was translated using Geneious Prime 2022.0.2 (https://www.geneious.com), and protein sequence comparisons were performed using BLASTx [34]. Conserved domain footprints in other putative protein-coding genes were identified using the National Center for Biotechnology Information Conserved Domains Database [35].

### Real-time RT PCR

Screening was performed using a real-time RT-PCR assay with TaqMan probes targeting the putative major capsid protein gene (MCP, accession no. MH061372) of SGPV (PoxMCP-F, CAG AGG TTT TTC ATA CGC CAG AA; PoxMCP-probe, TTA TAC ACC ATC ACA TTT GTG; PoxMCP-R, GAG GTC ACG GTG ATG ACA GAA C). As there is a higher copy number of mRNA molecules encoding the MCP than DNA molecules, targeting the RNA instead of DNA proved to be more sensitive. The specificity of the primers and probe was checked to ensure that they did not anneal with the host genome or with any poxvirid sequence other than that of SGPV. The assay was performed using an AgPath-ID™ One-Step RT-PCR Kit (Applied Biosystems) and run on a QuantStudio 3 Real-Time PCR System Cycler (Applied Biosystems). The combination of 400 nM forward and reverse primers and 225 nM probe gave the best result for the PoxMCP assay (efficiency = 1.968). The PCR efficiency was calculated from standard curves generated based on dilution series. Cycling conditions were 45 °C for 10 min and 95 °C for 10 min (reverse transcription step), followed by 45 cycles of 95 °C for 15 s and 60 °C for 45 s. During real-time RT-PCR on salmon tissues, an assay targeting elongation factor alpha from Atlantic salmon was used as internal control [36].

### Choice of variable regions for genotyping of SGPV

The central part of poxvirus genomes consists mainly of conserved genes that are essential for virus replication and exhibit little intraspecies variation. Genotyping based on these genes is not expected to permit variants of SGPV to be distinguished. However, it is known that the terminal parts of poxvirus genomes contain more variable regions that encode non-essential factors that affect virulence, host range, and immunomodulation [24]. In this study, several variable regions from both the central and terminal parts of the SGPV genome were identified by comparison of two SGPV genome sequences. One of these viruses was from Nordland County (KT159937), and the other was from Hordaland County (H2015/91, accession number OQ714485). The positions of the eight selected regions are shown in Table 1. These variables were tested with respect to intraspecies variation, ease of obtaining PCR products, and information provided during comparison of SGPV sequences from wild and farmed salmon from different locations.

**Table 1:** The selected variable (V) regions in SGPV and the percent nucleotide sequence identity between two SGPV isolates from Nordland (KT159937) and Hordaland (H2015/91) counties

Variable	KT159937	H2015/91 - KT159937	KT159937	H2015/91 – KT159937	% nt	Del.
V	CDS	Length ORF/INTG	Position	V length	identity	nt
V16	004 & 207	1428-1428	3361-4788 & 236777-238204	707-707	97.6	0
V26	012-013	-	11864-12657	793-794	98.0	1
V15	021-023	-	19416-20225	810-810	96.5	0
V27	040-041	-	34873-35619	747-747	97.9	0
V28	051	2718-2718	42950-45667	1340-1340	98.0	0
V29	073	1287-1287	63516-64802	699-699	97.0	0
V30	174	2562-2562	202382-204943	674-674	97.0	0
V5	191	972-960	220576-221535	499-487	95.9	12

The CDS (referring to KT159937), length of the ORFs or the position of intergenic variables also refers to KT159937. INTG, intergenic regions; %, similarity of the variable regions of the two SGPV isolates; Del, difference in the number of nucleotides in the compared regions

### PCR and sequencing

Each PCR reaction consisted of 23 μl of master mix and 2 μl of DNA or cDNA template. The amplification was based on a standard reaction mixture (16.0 µl of RNase-free water, 2.5 µl of 10x buffer, 2.5 µl of 1x dNTP (2.5 mM), 0.4 µM (1.0 µl) forward and reverse primers, and 0.5 µl of Taq DNA polymerase (5 U/µl). The primer pairs used to amplify selected variable regions and the predicted PCR products sizes are listed in Table 2. Three overlapping primer sets were designed to cover the entire length of V28 (1340 nt).

**Table 2:** Forward and reverse primers used to amplify eight selected variable regions

Variable	Primer sequence (5’-3’)		Product	Protein
	Primer forward	Primer reverse		
V16	TGCCATCCTCATCAAACTGACC	CCCGTTCATTGACTTCCTGATC	707	Hypothetical prot.
V26	GCGTGTTTATGTTCCATGCG	GGAGAGATATGTGACTCGTGTCTG	794	Hypothetical prot.
V15	ACCAGCCAATTTGTTCCG	ATTGCACAAGTGCCCGTG	810	Hypothetical prot.
V27	GAACATTCCCCCTACCGATAAC	GGTACATCCTGGAGTGTTGAAG	747	Ig domain type I membrane prot.
V28	GCCAACCATTACTGATTGCG	GGGACTCTTTATTGCTGTCTCTGG	648	Metalloendopeptidase
V28	GGCGGYTATGATGTATTCTC	ACAGAACTCTGTGGARTTGG	397	Metalloendopeptidase
V28	GTGGGTTTCCAAGTGATTGTCC	GGGACTCTTTATTGCTGTCTCTGG	714	Metalloendopeptidase
V29	GGCAAGTAGACTACAAGCACG	GAGTGACAGATCAGGACGG	698	Hypothetical prot.
V30	AGAATAGCCCACTGATCACC	GTACAACGGAAAGGACGG	699	Hypothetical prot.
V5	CCCGTTCATTGACTTCCTGATC	TGCCATCCTCATCAAACTGACC	707	Hypothetical prot.

The sizes of the PCR products refer to SGPV KT159937. The majority of the ORFs code for hypothetical proteins, while V28 and V27 are partial sequences of genes encoding a putative metalloendopeptidase and an Ig domain type I membrane protein, respectively. The identification of the proteins is based on BLAST search only

PCR was performed using an Applied Biosystems Veriti 96-Well Thermal Cycler (Thermo Fisher Scientific) at 94 °C for 2 minutes, followed by 35 cycles of 94 °C for 30 seconds, the annealing temperature of the specific primer pair for 30 seconds, and 72 °C for 1 minute, followed by one cycle of 72 °C for 10 minutes. The PCR products were stored at <4 °C.

The PCR products were visualized using gel electrophoresis. The gels were composed of 1.0 % Seakem LE Agarose dissolved in 1x Tris-acetate-EDTA (TAE) buffer and 1.0 μl of GelRed (Biotiom Inc). Two microliters of SmartLadder (Eurogentec) was added as a molecular weight marker. The gel was run for 10 minutes at 70 volts before increasing to 90 volts for 20-30 minutes. Ultimately, the gel was examined under UV light in a Gel Logic 212PRO gel imaging system (Fisher Scientific) with the program Carestream MI SE.

PCR products were subsequently purified by adding 2.0 μl of EXOSAP-IT (Affymetrix) to 5.0 μl of the post-PCR reaction mixture. The reaction was performed in an Applied Biosystems Veriti 96-Well Thermal Cycler at 37 °C for 15 minutes, followed by 80 °C for 15 minutes.

The purified products were sequenced by the Sanger method at the sequencing facility of the University of Bergen (http://www.uib.no/seqlab), using a Big Dye Terminator v3.1 Cycle Sequencing Kit (Applied Biosystems). The reaction mixture contained 1.0 μl of Big Dye 3.1 enzyme, 1.0 μl of Big Dye 5x Buffer, 3.2 pmol (1.0 μl) of forward and reverse primers, five to 20 ng of purified PCR product, and RNase-free water to 20 μl. The reactions were run using a Biosystems Vereti 96-Well Thermal Cycler with the standard sequencing program. The cycling conditions were 5 minutes at 96 °C followed by 25 cycles of 96 °C for 10 minutes, 56 °C for 5 seconds, and 60 °C for 4 minutes. Unincorporated dye terminators and salt ions from the extension cycle were removed using a D-Pure™ DyeTerminator Cleanup Kit (Nimagen). Sanger DNA sequencing was performed using a capillary-based Applied Biosystems 3730XL Analyzer.

### Phylogenetic analysis based on selected variable regions

Selected sequences from other members of the family *Poxviridae* obtained from the EMBL nucleotide database were included in alignments of selected putatively conserved genes. The amino acid sequences of four putative proteins from SGPV (H2015/91), including mRNA capping enzyme large subunit (accession no. MH061373), DNA-directed RNA polymerase subunit alpha (accession no. MH061370), P4B major core protein (accession no. MH061371), and major capsid protein (accession no. MH061372), were aligned with their counterparts from other poxviruses. Members of the subfamily *Entomopoxvirinae* were excluded because of their large amino acid sequence divergence from SGPV. Ambiguously aligned regions were removed using Gblocks [36]. Phylogenetic relationships were determined using the maximum-likelihood (ML) method in TREE_PUZZLE 5.2 (available at: http://www.tree-puzzle.de), employing the VT model of amino acid substitution [37].

The sequences of the variable regions of the SGPV genome were preliminarily identified by GenBank searches done with BLAST 2.0 [34], and the Vector NTI Suite software package was used to make multiple alignments. To perform pairwise comparisons of the sequences, the multiple sequence alignment editor GeneDoc (available at: https://nrbsc.org/gfx/genedoc/) was used for manual adjustment of the sequence alignments.

Nucleotide sequence alignments edited in GeneDoc were used for phylogenetic analysis based on the selected variable regions in the SGPV genome. The substitution models for the eight variables were calculated using J-Modeltest [38]. Phylogenetic relationships were determined using the maximum-likelihood (ML) method available in TREE_PUZZLE 5.2 (available at http://www.tree-puzzle.de). Quartet puzzling was used to choose from the possible tree topologies and to simultaneously infer support values for internal branches. Quartet trees were based on approximate maximum likelihood values using the selected model of substitution and rate heterogeneity. The robustness of each node was determined using 50,000 puzzling steps. Phylogenetic trees were drawn using TreeView [39]. The GenBank accession numbers of the sequences from this study are presented in ESM_1.

## Results

### Partial genome sequence of SGPV (H2015/91)

**Fig. 2:**
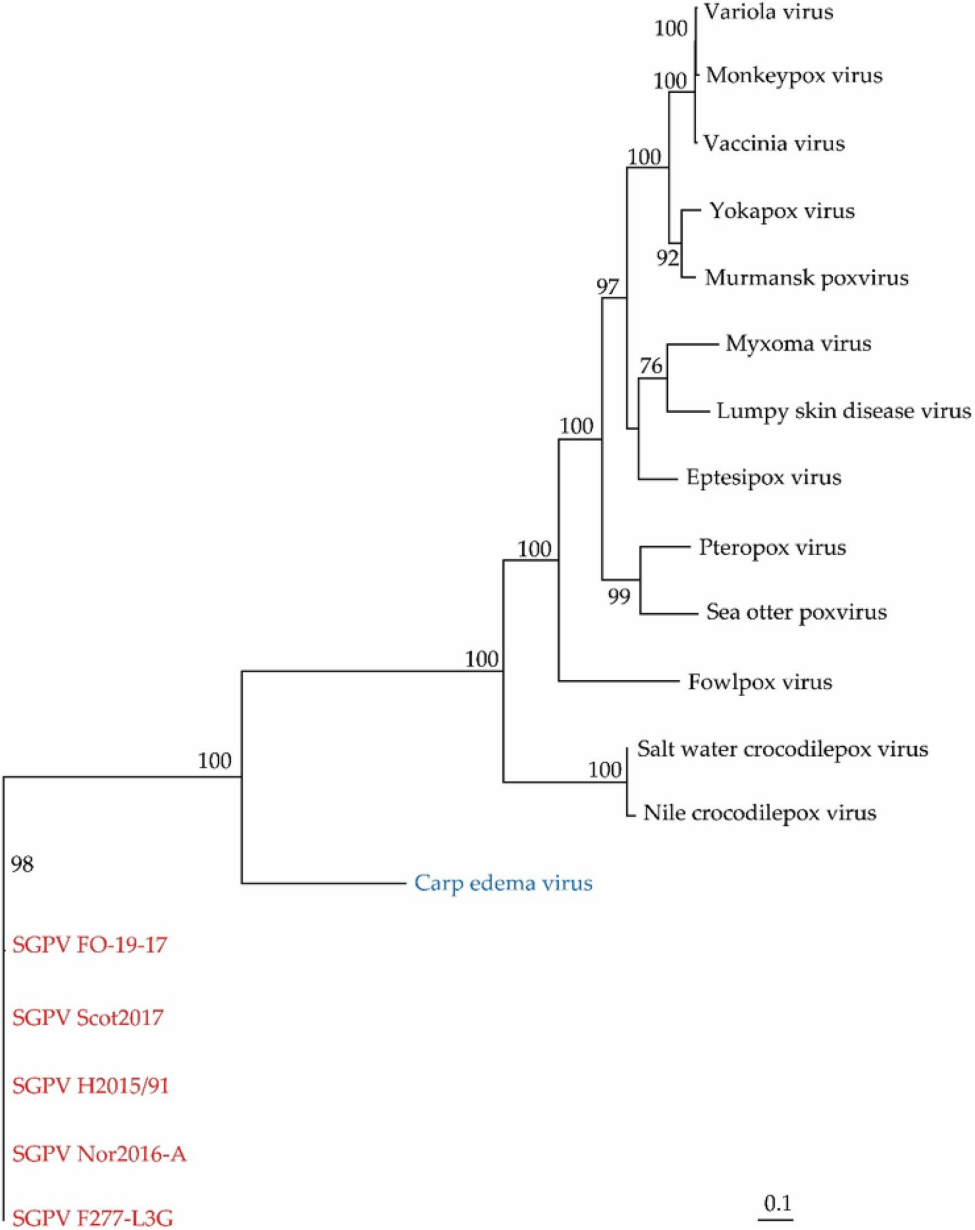
Phylogenetic positions of five SGPV isolates in relation to selected chordopoxviruses based on analysis of major capsid protein (MCP) sequences after removal of ambiguously aligned regions using Gblocks. Branch lengths represent relative phylogenetic distances according to maximum-likelihood estimates based on the VT matrix. Colored labels indicate the origin of the fish: wild fish in freshwater, green; wild fish in seawater, blue; farmed fish in freshwater, purple; farmed fish in seawater, red. W, wild fish

Illumina sequencing of SGPV H2015/91 from Hordaland County gave a total of 56,397,192 reads (average coverage, 74.11), yielding 172,263 scaffolds (70,783,900 bp) with an average quality (Phred) score of 35.1. The generated contigs identified as putative SGPV sequences were aligned with the genome sequence of an SGPV isolate from Nordland County (KT159937, 2012-04-F277-L3G). The partial genome sequence of H2015/91, consisting of 240,954 bp, was identified, and gaps were closed by Sanger sequencing (accession no. OQ714485). The phylogenetic position of H2015/91 was determined by phylogenetic analysis based on four conserved genes (major capsid protein MH061372, major core protein MH061371, DNA-directed RNA polymerase subunit A MH061370, and mRNA capping enzyme large subunit MH061373). Phylogenetic analysis, based on the major capsid protein showed that SGPV isolates from Norway, Scotland, and the Faeroe Islands are distantly related to other chordopoxviruses, with carp edema virus (CEV) from koi carp (*Cyprinus carpio*) as the closest relative (Fig. 2). Only members of the subfamily *Chordopoxvirinae* were included in the analysis. Members of the subfamily *Entomopoxvirinae* were excluded due to low amino acid sequence similarity to SGPV. The nucleotide sequence identity of 72 ORFs of H2015/91 and KT159937 coding for previously identified proteins ranged from 97.9 to 100.0%.

### Variable regions suitable for genotyping of SGPV

The work on identifying suitable variable regions for genotyping of SGPV started with a large number of variable regions, both within ORFs and in intergenic regions. As expected, there was more variation in the ORFs in the terminal parts of the genome than in the central part. Variable numbers of tandem repeats (VNTRs) were present in some of the intergenic regions, but not in all. The VNTR regions can be used to distinguish SGPV variants but are not suitable for phylogenetic analysis based on homolog sequences. Moreover, VNTRs might evolve independently by homoplasy and thus interfere with accurate phylogenetic reconstruction. Three of the intergenic variable regions (V15, V26, and V27) that did not contain any tandem repeats were used in this study, while those containing VNTRs were excluded. Five variable regions within ORFs (V5, V16, V28, V29, and V30) were included in the genotyping system, as they provided valuable information for phylogenetic analysis. The variation within all of these ORFs except for V5 was the result of nucleotide substitutions. V5 contained tandem repeats within the ORF, resulting in gaps in the alignment, which, however, were removed before the analysis. The eight variable regions were selected based on intraspecies variation, the absence of frequent deletions, the ability to provide phylogenetic information, and the ease of obtaining a PCR product. The primer pairs used for amplification of these regions are listed in Table 2.

SGPV is present in wild and farmed salmon in all counties in Norway. However, real-time RT-PCR (MCP assay) screening of gill tissues from wild salmon in most cases yielded Ct values above 27.0, making it difficult to obtain sequence information. Since the SGPV sequences used in this study were selected based on their Ct values (< 27) in the MCP assay, they may not be completely representative of the natural variation of SGPV in Norway.

### Selected variable regions

V16 is present in two ORFs, CDS004 and 207 (both hypothetical proteins containing two low-complexity regions in the putative proteins), one at each end of the SGPV genome. The length of the product used in phylogenetic analysis was 663 bp. All 74 SGPV sequences included in the analysis of this region had 93.8% of their nucleotides in common. No deletions or insertions were found in this region.

V26 covers an intergenic region including nucleotides from two ORFs (CDS012-013). The length of the product used in phylogenetic analysis was 728 bp, and the 42 SGPV sequences included in the analysis of this region had 94.9% of their nucleotides in common. No deletions or inserts were found in this region.

V15 covers a sequence spanning an intergenic region and includes parts of three ORFs (CDS021-023). The length of the sequence used in the analysis was 774 bp. The 61 SGPV sequences used in the analysis of this region had 93.4% of their nucleotides in common. No deletions or insertions were found in this region.

V27 covers a sequence spanning an intergenic region and includes parts of CDS040-041. The length of the product used in phylogenetic analysis was 747 bp. CDS040 encodes a putative Ig domain type I membrane protein. The 37 SGPV sequences used in the analysis of this region had 93.6% of their nucleotides in common. No deletions or insertions were found in this region.

V28 is the largest variable region included in this study and consists of 1329 bp in CDS051, which encodes a putative metalloendopeptidase (with two low-complexity regions in the putative protein). The 59 SGPV used in the analysis of this region had 95.4% of their nucleotides in common. No deletions or insertions were found in this region.

V29 includes 676 bp inside CDS073, which encodes a hypothetical protein containing two low-complexity regions. The 60 SGPV sequences used in the analysis of this region had 95.4% of their nucleotides in common. No deletions or insertions were found in this region.

V30 includes 674 bp in CDS174, which encodes a hypothetical protein. The 28 SGPV used in the analysis of this region had 96.4% of their nucleotides in common. No deletions or insertions were found in this region.

V5 is present in CDS191, which encodes a hypothetical protein with two low-complexity regions and covers 453-477 bp. The length of the product used in phylogenetic analysis was 453 bp after deletion of gaps in the sequences (24 bp). The 49 SGPV sequences included in the analysis of this region had 91.4% of their nucleotides in common after removal of the gaps. Repeated sequences (12 nucleotides long) were present within this ORF. The number of repeats and the nucleotide composition of the 12 nucleotides constituting the repeats differed among the different strains. Nine different repeats were observed among the strains analyzed: AGTGATCTTGAC, AGTGATCAAGAC, AGTGACCTTGAC, AGTGACCAAGAC, AGTGATCTAGAC, AGTGATCATGAC, AGTGACCAGGAA, AGTGATCAGGAA, and AGTGACCATGAA.

### Prevalence of SGPV in wild Atlantic salmon in Norway

In Norway, SGPV is relatively common in farmed smolt in fresh water and salmon at marine production sites, where it can be associated with mortality (Nylund, personal observation). The mortality in sea water usually occurs in concert with a range of other gill pathogens, including *Paramoeba perurans*, *Paranucleospora theridion*, *Ichthyobodo* spp., and *Candidatus* Branchiomonas cysticola. The prevalence of SGPV in farmed salmon varies from 0 to 100%.

The wild salmon and trout included in this study were collected at marine sites in counties with intensive salmon farming and from rivers emptying into fjords in these counties. Five of the fjords, Vestfold, Trondheimsfjord, Namsfjord, Altafjord, and Kongsfjord, have the status of protected National Salmon Fjords, but around the opening of the four latter fjords are high densities of salmon farms. It is expected that wild salmon returning to these coastal areas could experience a significant infection pressure due to the high density of farmed salmon. However, the data from the wild salmon, collected using fish traps at sea sites, show a relatively low prevalence of the virus – 0.0% (Kongsfjord)-13.2% (Kvaløya) – while the prevalence of the virus in salmon from the rivers is higher in nearly all study areas (Table 3). The prevalence of SGPV at Agdenes, at the mouth of Trondheimsfjord, is 4.4%, while the prevalence in the rivers emptying into this fjord range from 5.9% (Stjørdal River) to 21.8% (Orkla and Steinkjær rivers). In the other fjord system in northern Trøndelag, the prevalence of SGPV is 2.5% in Namsfjord, while the prevalence in Namsen and the upper Namsen River (Sæter-Namsen) is 16.2 % and 54.2%, respectively. A similar pattern can be seen in fjords and rivers in Finnmark County (Table 3).

**Table 3:** Prevalence of SGPV in wild Atlantic salmon (*Salmo salar*) collected at sea sites (fjords) and associated river systems

Location	N	Pos	%	Code	Year	Geographical location
Skjern* River	18	18	100	DK	2010	Denmark
Vestfold	439	32	7.3	V	2019-2022	Vestfold County
Lyse River	174	42	25.3	R	2007-2022	Rogaland County
Etne* River	21	21	100	H	2009	Hordaland County Hardanger fjord
Etne River	47	11	23.4	H	2010	Hordaland County Hardanger fjord
Sørfjord	93	42	46.7	H	2009-2011	Hordaland County, Sørfjord
Vosso* River	32	18	56.3	H	2012	Hordaland County, Sørfjord
Dale* River	55	47	85.5	H	2009-2013	Hordaland County, Sørfjord
Gaular River	26	8	30.8	SF	2015	Sogn & Fjordane County
Agdenes	1084	47	4.3	ST	2014-2022	Trøndelag, Trondheimsfjord
Orkla River	265	58	21.8	ST	2013-2017	Trøndelag, Trondheimsfjord
Gaula River	568	77	13.6	ST	2013-2022	Trøndelag, Trondheimsfjord
Stjørdal River	576	34	5.9	NT	2013-2022	Trøndelag, Trondheimsfjord
Steinkjær River	133	29	21.8	NT	2016-2020	Trøndelag, Trondheimsfjord
Kvaløy	337	47	13.9	NT	2018-2021	Trøndelag
Namsfjord	748	19	2.5	NT	2015-2022	Trøndelag, Namsfjord
Namsen River	1090	177	16.2	NT	2013-2022	Trøndelag, Namsfjord
Sæter-Namsen River	227	123	54.2	NT	2013-2022	Trøndelag, Namsfjord
Altafjord	490	7	1.4	F	2017-2022	Finnmark County, Altafjord
Alta River	553	190	34.4	F	2016-2022	Finnmark County, Altafjord
Kongsfjord	31	0	0.0	F	2020	Finnmark County, Kongsfjord
Kongsfjord River	152	16	10.5	F	2020-2022	Finnmark County, Kongsfjord

All locations are in Norway except for the Skjern River, located in Denmark. N, number of salmon tested; Pos, number of salmon positive for SGPV; %, the prevalence of SGPV in salmon. Sæter Namsen is the upper part of Namsen river

*Salmon broodfish were kept in tanks before sampling

The data obtained from wild salmon in the sea in western Norway give a slightly different picture from the observations in Trøndelag and Finnmark. The prevalence of SGPV in Sørfjord (Hordaland County) was as high as 46.7% (N = 93). This fjord has an upper layer of brackish water and is used for production of rainbow trout (*Oncorhynchus mykiss*) only, i.e., a species that does not carry SGPV. The prevalence of SGPV in the Lyse River (Rogaland County) and the rivers in Hordaland (Etne, Dale, and Vosso) ranged from 23.4% (Etne 2010) to 25.3% (Lyse) and was 100% during sampling of the Etne in 2009. However, those samples were from broodfish kept in tanks after being moved from rivers, and the high prevalence could therefore be a result of horizontal transmission within the tanks. Salmon collected from the Etne River in 2010 had a prevalence of infection of 23.4%. Salmon from the Gaular River in Sogn and Fjordane County had a prevalence of SGPV of 30.8%. The majority of the SGPV-positive wild salmon had low viral loads, i.e., Ct values above 27. Since all of the sequences used in this study were obtained from the gills of salmon with Ct values <27, it is not known if this initial selection excluded some variants of SGPV.

The 405 trout (*Salmo trutta*) collected from rivers and from the sea in Trøndelag and Finnmark were all negative for the presence of SGPV.

### Relationships between SGPV isolates from farmed and wild salmon

**Fig. 3:**
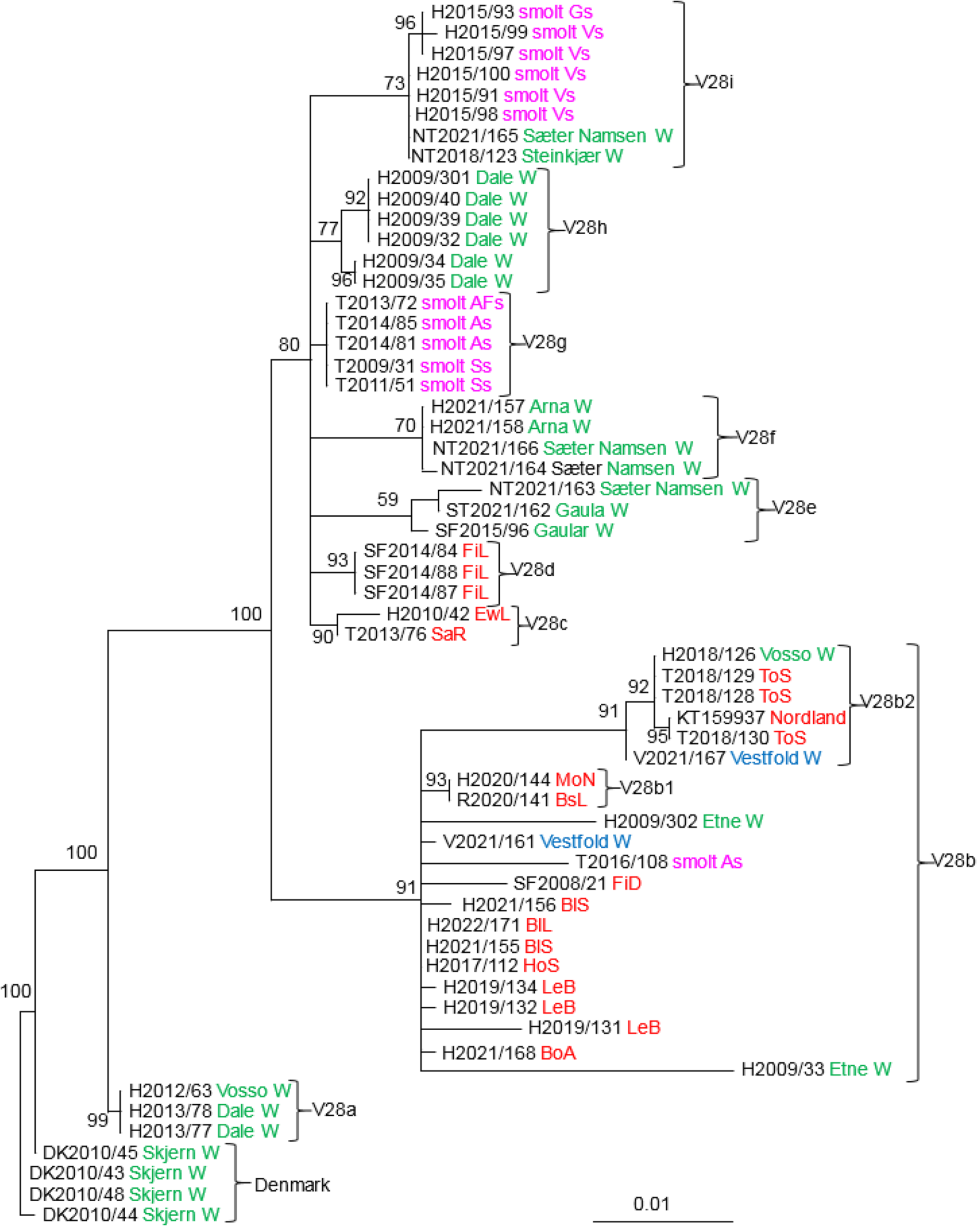
Phylogenetic relationships among SGPV isolates from farmed and wild salmon (N = 59) based on variable region V28. A maximum-likelihood quartet puzzling tree is shown. The best-fitting nucleotide substitution model (GTR) was used for maximum-likelihood analysis, and the tree was bootstrapped (50,000 quartet puzzling steps) using TREE_PUZZLE. Branch lengths represent relative phylogenetic distances based on maximum-likelihood estimates. Colored labels indicate the origin of the fish: wild fish in freshwater, green; wild fish in seawater, blue; farmed fish in freshwater, purple; farmed fish in seawater, red. W, wild fish

**Fig. 4:**
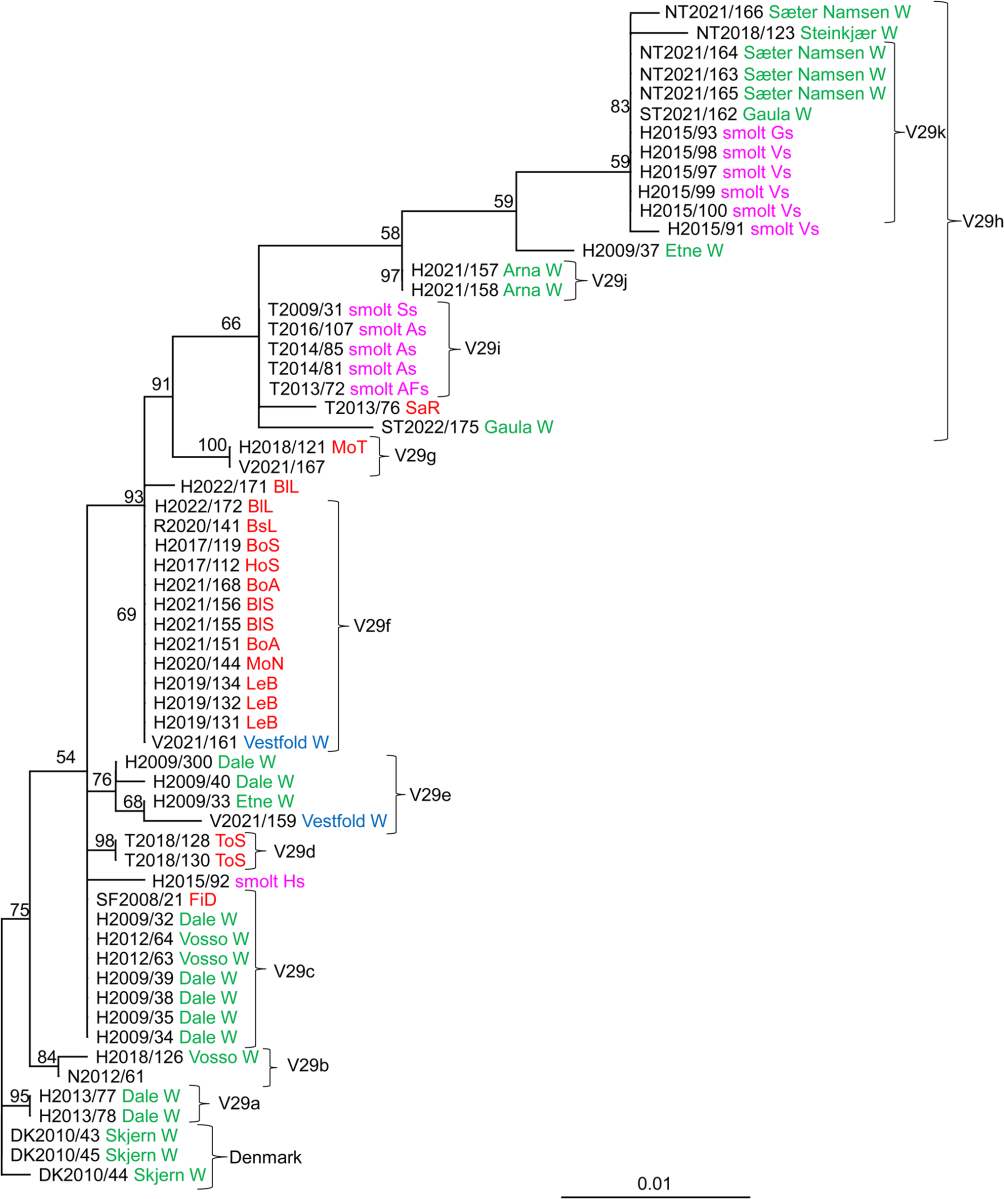
Phylogenetic relationships between SGPV isolates from farmed and wild salmon (N = 60) based on variable region V29*.* A maximum-likelihood quartet puzzling tree is shown. The best-fitting nucleotide substitution model (GTR) was used for maximum-likelihood analysis, and the tree was bootstrapped (50,000 quartet puzzling steps) using TREE_PUZZLE. Branch lengths represent relative phylogenetic distances based on maximum-likelihood estimates. Colored labels indicate the origin of the fish: wild fish in freshwater, green; wild fish in seawater, blue; farmed fish in freshwater, purple; farmed fish in seawater, red. W, wild fish

**Fig. 5:**
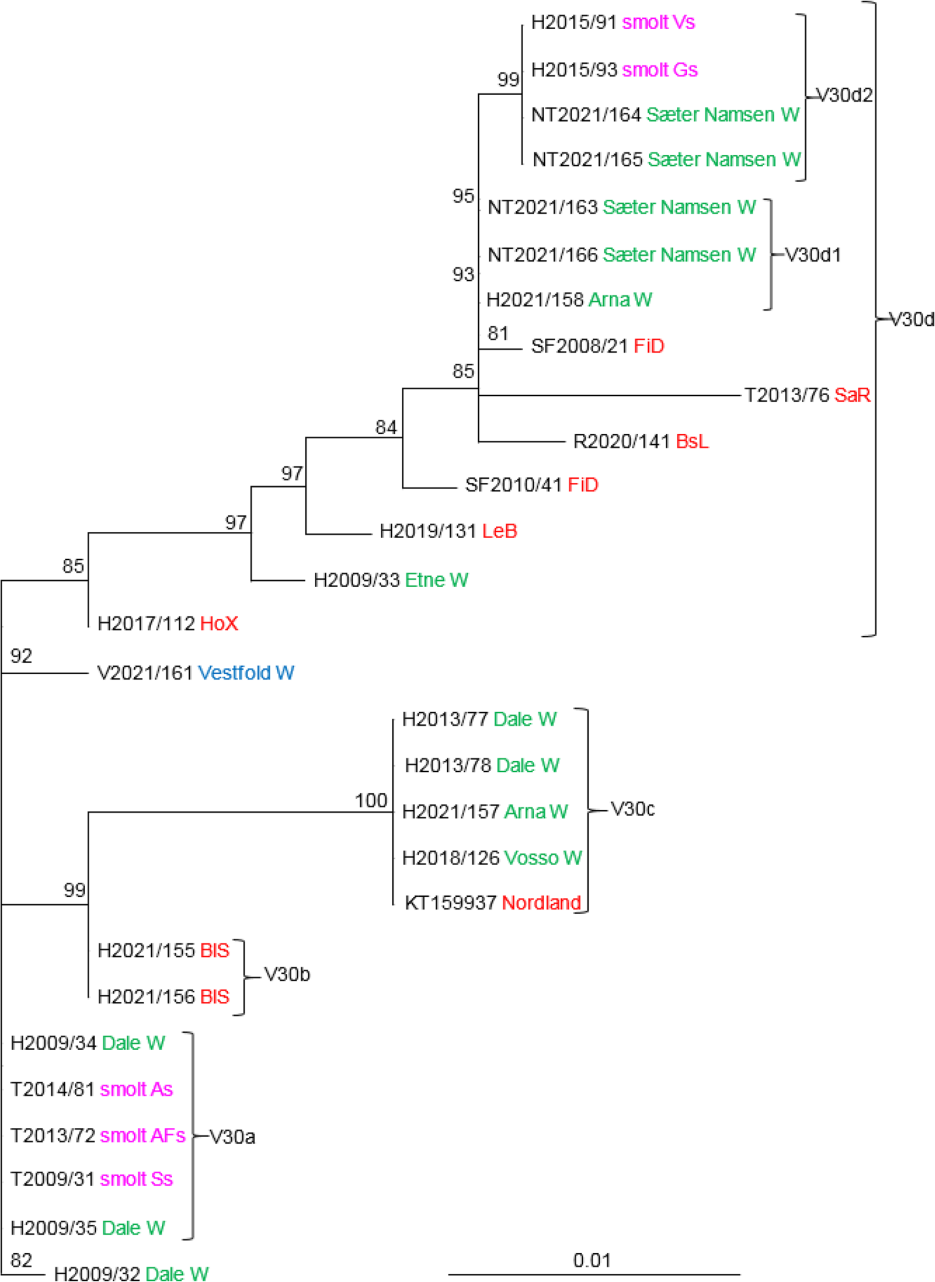
Phylogenetic relationships among SGPV isolates from farmed and wild salmon (N = 28) based on variable region V30. A maximum-likelihood quartet puzzling tree is shown. The best-fitting nucleotide substitution model (GTR) was used for maximum-likelihood analysis, and the tree was bootstrapped (50,000 quartet puzzling steps) using TREE_PUZZLE. Branch lengths represent relative phylogenetic distances based on maximum-likelihood estimates. Colored labels indicate the origin of the fish: wild fish in freshwater, green; wild fish in seawater, blue; farmed fish in freshwater, purple; farmed fish in seawater, red. W, wild fish

**Fig. 6:**
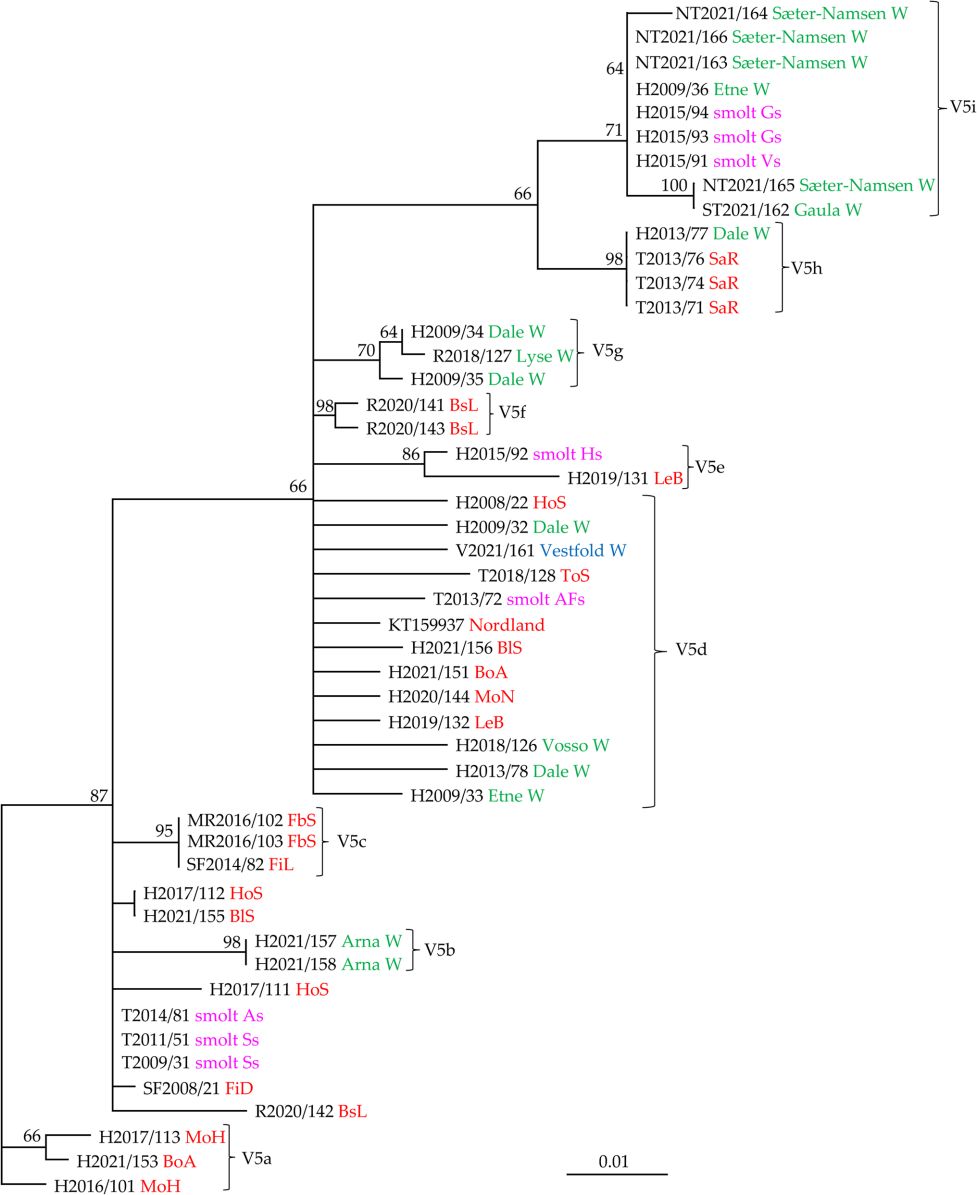
Phylogenetic relationships among SGPV isolates from farmed and wild salmon (N = 49) based on variable region V5. A maximum-likelihood quartet puzzling tree is shown. The best-fitting nucleotide substitution model (GTR) was used for maximum-likelihood analysis, and the tree was bootstrapped (50,000 quartet puzzling steps) using TREE_PUZZLE. Branch lengths represent relative phylogenetic distances based on maximum-likelihood estimates. Colored labels indicate the origin of the fish: wild fish in freshwater, green; wild fish in seawater, blue; farmed fish in freshwater, purple; farmed fish in seawater, red. W, wild fish

The phylogenetic relationships between SGPV isolates from farmed and wild salmon, based on analysis of variable regions V28, V29, V30, and V5 indicated the existence of several distinct clades with good support values (Figs. 3, 4, 5, 6). The analysis of V28, which included 59 SGPV isolates, resulted in nine clades, in addition to a group consisting of SGPV isolates from the Skjern River in Denmark (Fig. 3). Clades V28b and V28i both include viruses from farmed and wild salmon, while clades V28a, V28e, V28f, and V28h include SGPV from wild salmon only. The three viruses in clade V28a are identical and were collected from two neighboring rivers in the same fjord system in 2012-2013. Differences at two nucleotide positions were seen among the SGPV isolates collected from Skjern, Denmark, in 2010. Clade V28b includes 21 isolates collected in the period 2008-2022 from both farmed and wild salmon in fresh and sea water. These viruses were collected from six different counties (Vestfold, Rogaland, Hordaland, Sogn and Fjordane, Nordland, and Troms), covering most of the Norwegian coastline. Two viruses collected in Vestfold, an area without salmon farming, grouped in this clade with close relationships to viruses from farmed salmon in the sea. A substitution at one nucleotide position separated V2021/161 from three viruses from Hordaland County (H2017/112, H2021/155, and H2021/171). All of the members of clade V28g were collected from farmed salmon in fresh water (smolt production sites), while the viruses in clade V28i were collected from farmed salmon smolt and wild salmon in rivers. Viruses collected from the smolt production site Ss (T2009/31 and T2011/51) were identical despite being collected in 2009 and 2011 (V28g). These viruses were also identical to those collected from two other smolt production sites (As and AFs) in Troms County. Three out of five SGPV isolates sequenced from five salmon at site Vs in 2015 (V28i) were identical, while the other two differed at one and two nucleotide positions. The three identical viruses from site Vs (Hordaland) were also identical to two viruses collected from the rivers Steinkjær and Sæter Namsen in 2018 and 2021. SGPV collected from six salmon in the Dale River (V28h) were identical except for two (H2009/34 and H2009/35) that differed at three positions. These six salmon were kept in the same tank before sampling, and the high level of sequence similarity could be a result of horizontal transmission within the tank. Two viruses collected from salmon in the Arna River in Hordaland were identical to a virus collected in the Sæter-Namsen River in Trøndelag (V28f). Five of the viruses in V28c and V28d were collected from farmed salmon in the sea and formed two well-supported clades.

The analysis of V29 included 60 SGPV isolates that grouped into several distinct clades (Fig. 4). Identical viruses from smolt production sites were also confirmed in the analysis of this variable region. Five viruses collected at three smolt sites in Troms in the period 2009-2016 were identical (V29i), and five viruses collected at sites Vs and Gs in Hordaland (2015) were identical (V29k). The sixth virus (H2015/91) from site Vs differed at only one nucleotide position. The five viruses (Vs and Gs) were also identical to four viruses collected from two different rivers in Trøndelag (Gaula and Sæter-Namsen). Identical viruses were also found in two neighboring rivers in Hordaland County; Vosso (2012) and Dale (2009) (V29c). These viruses were also identical to an isolate (SF2008/21) from farmed salmon in Sogn og Fjordane County. Three viruses from the Skjern River (Denmark) were closely related to two viruses from the Dale River (V29a), two of them being different at only one nucleotide position. Clade V29e contains viruses exclusively from wild salmon in Hordaland (collected in 2009) and Vestfold (V2021/159). Twelve isolates from marine salmon production (2017-2022) in western Norway (Rogaland and Hordaland) were identical to a virus collected from wild salmon in Vestfold (2021), i.e., an area on the east coast of Norway without any salmon farming (V29f).

Only 28 isolates were included in the analysis of V30 (Fig. 5). Clade V30a consisted of five identical viruses collected in 2009-2014 from fresh water; at smolt production sites (Troms: Ss, As, and AFs) and the Dale River (Hordaland). These viruses differed at only one nucleotide position from another virus obtained from wild salmon in the Dale River (H2009/32). Another distinct clade of identical SGPV isolates, V30c, consists of viruses from three rivers in Hordaland and one marine farm in Nordland County. These viruses were collected in the period from 2012 (KT159937) to 2021 (H2021/157). Clade V30d consists of a diverse well-supported group of isolates from both rivers and production sites for farmed salmon in both freshwater and seawater. Sub-clade V30d1 consists of identical viruses from wild salmon in western and central Norway, while V30d2 contains identical viruses from wild salmon in the Sæter River Namsen and from smolt at two different production sites in Hordaland County.

Analysis of V5 grouped the SGPV isolates (N = 49) into several clades in addition to viruses with uncertain positions with respect to the supported clades (Fig. 6). Three clades include viruses exclusively from farmed salmon in seawater (V5a, V5c, and V5f), and another three clades contain viruses from salmon in both freshwater and seawater (V5d, V5e, and V5h). Analysis of this variable showed, as seen above, identical viruses collected in different years from the same smolt sites and from different smolt sites (Ss-As, and Vs-Gs). SGPV isolates from wild salmon were present in five clades (V5b, V5d, V5g, V5h, and V5i). Five viruses from wild salmon in Hordaland County and Vestfold (V2021/161) were in uncertain positions with respect to the viruses from farmed salmon in V5d.

**Fig. 7:**
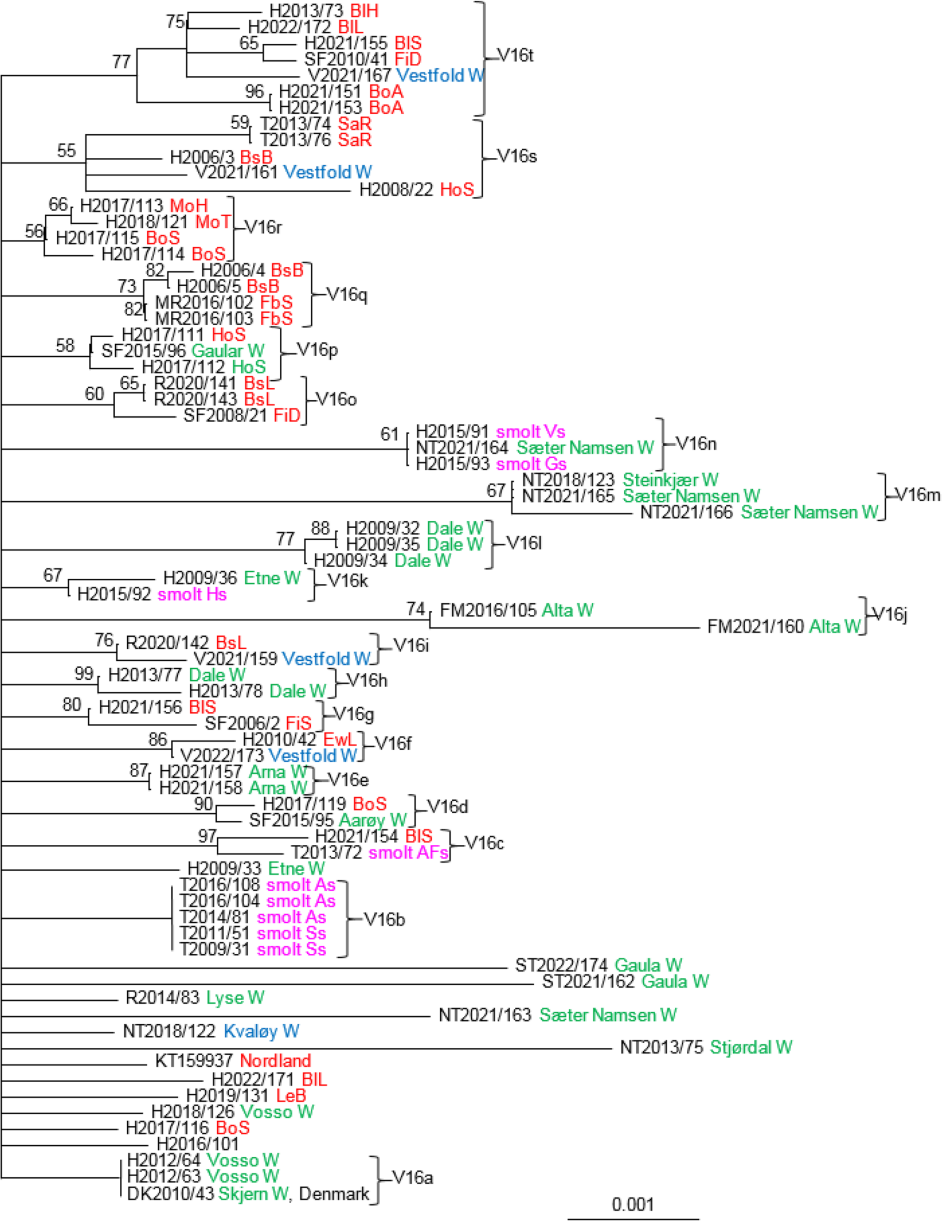
Phylogenetic relationship among SGPV isolates from farmed and wild salmon (N = 74) based on variable region V16. A maximum-likelihood quartet puzzling tree is shown. The best-fitting nucleotide substitution model (GTR) was used for maximum-likelihood analysis, and the tree was bootstrapped (50,000 quartet puzzling steps) using TREE_PUZZLE. Branch lengths represent relative phylogenetic distances based on maximum-likelihood estimates. Colored labels indicate the origin of the fish: wild fish in freshwater, green; wild fish in seawater, blue; farmed fish in freshwater, purple; farmed fish in seawater, red. W, wild fish

V16 is present in two separate ORFs (CDS004 and CDS207), one at each end of the SGPV genome. We did not find any differences in V16 from the two ORFs within the genome of SGPV from individual salmon. The analysis of this variable region included isolates from 74 salmon collected in both freshwater and seawater (Fig. 7). The observed variation was high, and the support values for the different clades were low, with a few exceptions. Isolates collected in the period from 2009 to 2016 from two smolt facilities (group V16b) in Troms County were identical. Two viruses collected in the Vosso River were also identical to a virus collected from salmon in the Skjern River in Denmark (V16a). The rest of the viruses differed from each other, with a few exceptions; two viruses collected from each of the marine salmon farms BoA (V16t), SaR (V16s), FbS (V16q), and BsL (V16o) and two viruses from wild salmon collected in the rivers Arna (V16e) and Dale (V16l) were identical. A third virus (H2009/34) collected from salmon in the Dale the same year differed at only one nucleotide position. The marine farm BoS was stocked with smolt from two different smolt production sites in the spring of 2017. The smolt originated from two different broodstocks; one in Hordaland and another in Møre and Romsdal. Gills were sampled from salmon in the autumn of 2017. Four isolates from salmon at the marine site, three (H2017/115, H2017/114, and H2017/119) originating from smolt produced in Hordaland, and one (H2017/116) from smolt originating from Møre and Romsdal were sequenced. Analysis of the four isolates collected from farm BoS showed that they were all distinct strains. Three of the viruses grouped with other viruses (V16d and V16r), while the fourth (H2017/116) had an unresolved position. The diversity of SGPV on this farm could suggest horizontal transmission of the virus from wild salmon or surrounding salmon farms.

**Fig 8:**
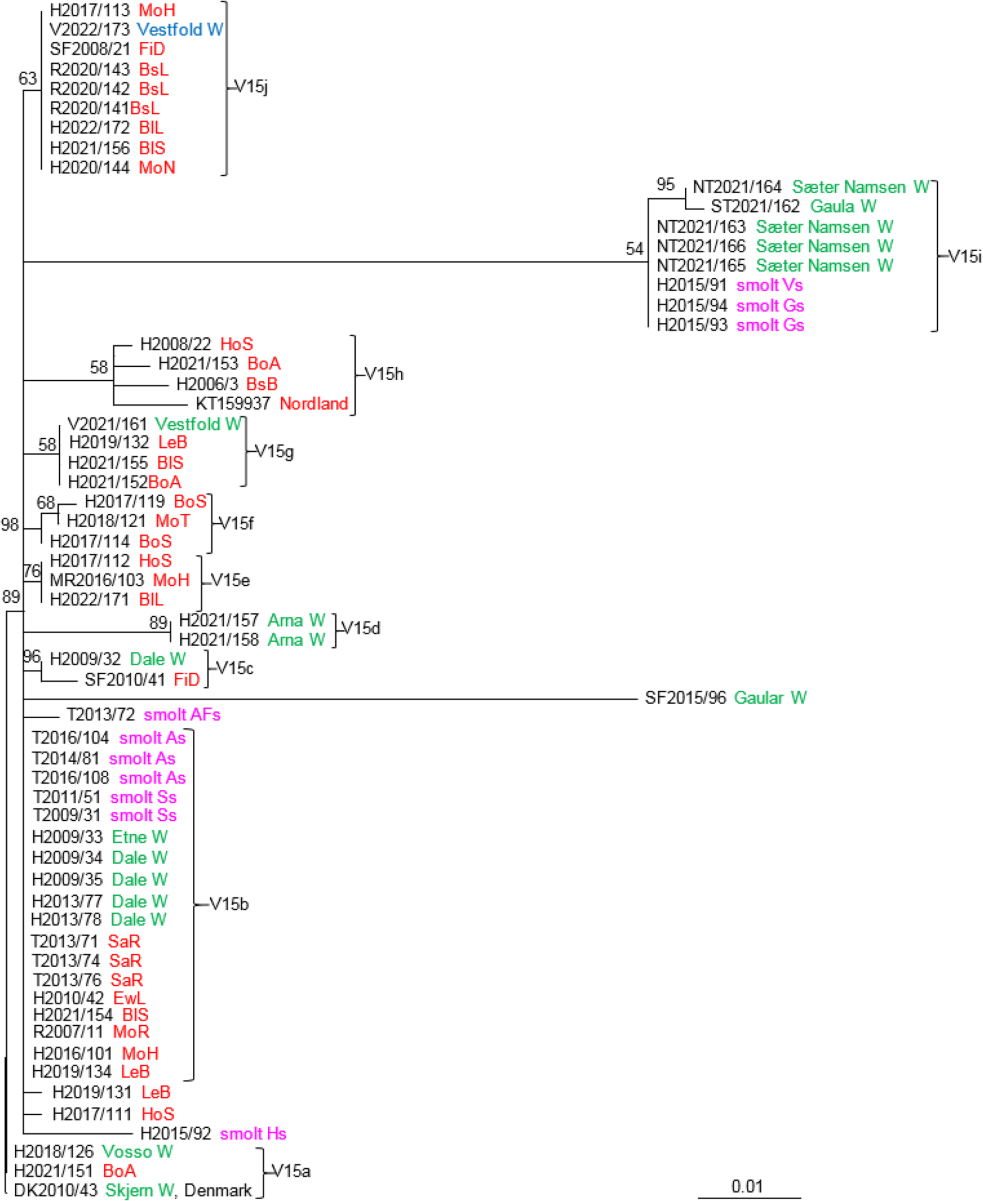
Phylogenetic relationships among SGPV isolates from farmed and wild salmon (N = 61) based on variable region V15. A maximum-likelihood quartet puzzling tree is shown. The best-fitting nucleotide substitution model (GTR) was used for maximum-likelihood analysis, and the tree was bootstrapped (50,000 quartet puzzling steps) using TREE_PUZZLE. Branch lengths represent relative phylogenetic distances based on maximum-likelihood estimates. Colored labels indicate the origin of the fish: wild fish in freshwater, green; wild fish in seawater, blue; farmed fish in freshwater, purple; farmed fish in seawater, red. W, wild fish

The remaining three variable regions, V15, V26, and V27, include sequences between ORFs but do not contain any tandem repeats. The analysis of V15 included SGPV isolates collected from 61 individual salmon. Ten groups were identified, including four well-supported clades (V15a, V15c, V15d, and V15e) (Fig. 8). Group V15b includes 18 identical viruses collected from both fresh and seawater salmon farming, one virus from the Etne River and four viruses from the Dale River sampled in 2009 and 2013. The viruses from farmed salmon were collected in Troms, Hordaland, and Rogaland in the period 2007-2021. A virus from the Skjern River in Denmark (DK2010/43) was identical to one from the Vosso River (H2018/126) and one from the marine salmon farm BoA (H2021/151) (V15a). Three viruses from smolt sites in Hordaland were identical to three viruses from a river in Trøndelag (V15i). V15j consists of nine identical isolates: eight from farmed salmon in the sea in three different counties in western Norway and one from wild salmon collected in Vestfold County.

**Fig. 9:**
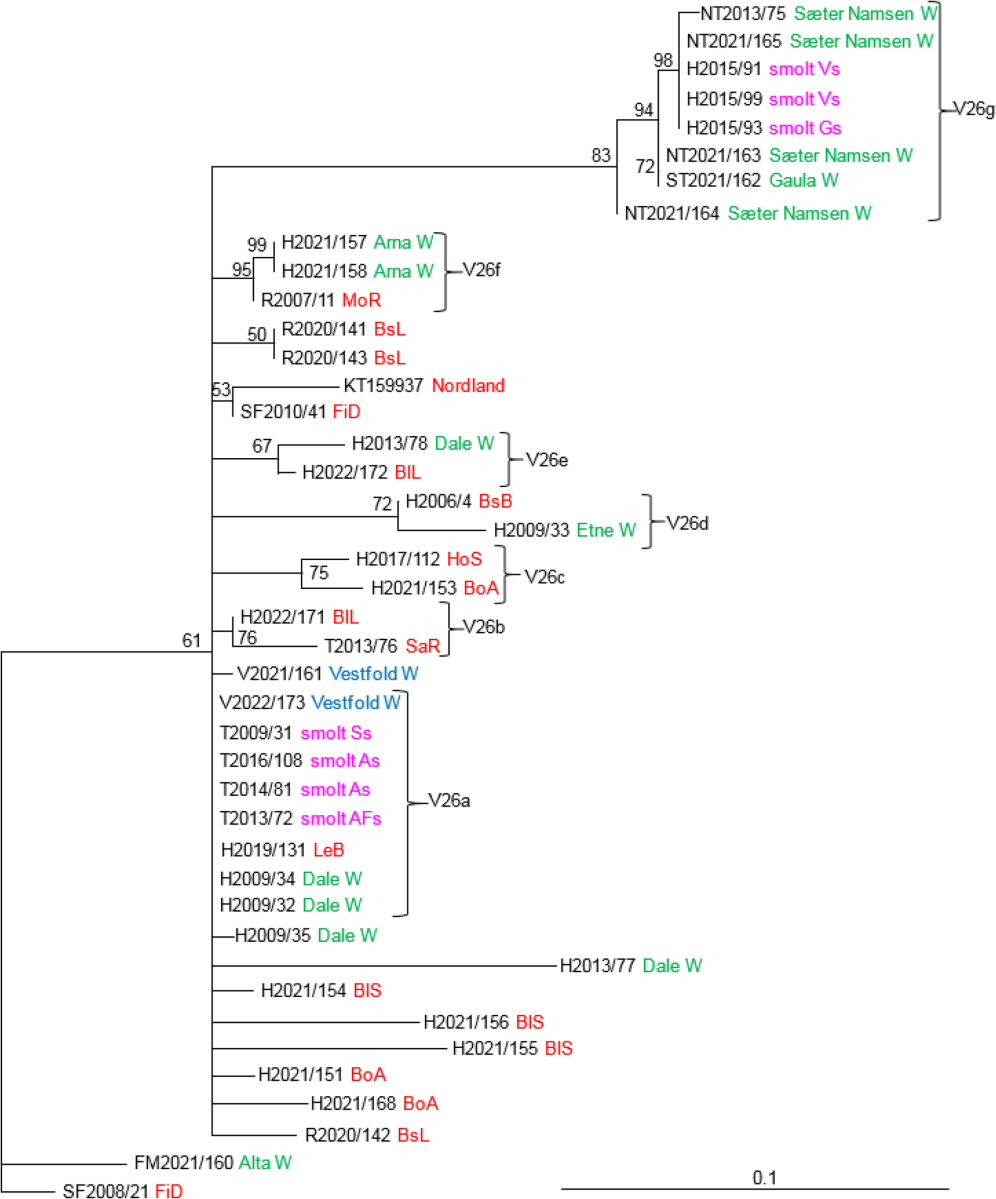
Phylogenetic relationships among SGPV isolates from farmed and wild salmon (N = 42) based on variable region V26*.* A maximum-likelihood quartet puzzling tree is shown. The best-fitting nucleotide substitution model (GTR) was used for maximum-likelihood analysis, and the tree was bootstrapped (50,000 quartet puzzling steps) using TREE_PUZZLE. Branch lengths represent relative phylogenetic distances based on maximum-likelihood estimates. Colored labels indicate the origin of the fish: wild fish in freshwater, green; wild fish in seawater, blue; farmed fish in freshwater, purple; farmed fish in seawater, red. W, wild fish

The analysis of V26 included 42 SGPV isolates. The resulting phylogeny resulted in a few well-supported clades (Fig. 9). Viruses from farmed and wild salmon group together in five groups (V26a, V26d, V26e, V26f, and V26g). Clade V26g consists of only isolates from freshwater and includes three identical viruses collected in 2015 from two different smolt facilities (Vs and Gs) in Hordaland County. Vs had delivered salmon fry to Gs, which could explain the similarity. SGPV from smolt collected in the period 2009-2016 in As, Ss, and AFs in Troms County were also identical (group V26a), but there are no known connections between these smolt facilities. These viruses are also identical to two viruses collected from salmon in the Dale River in 2009, one virus from wild salmon in Vestfold, and one on a marine farm in Hordaland (H2019/131 LeB).

**Fig. 10:**
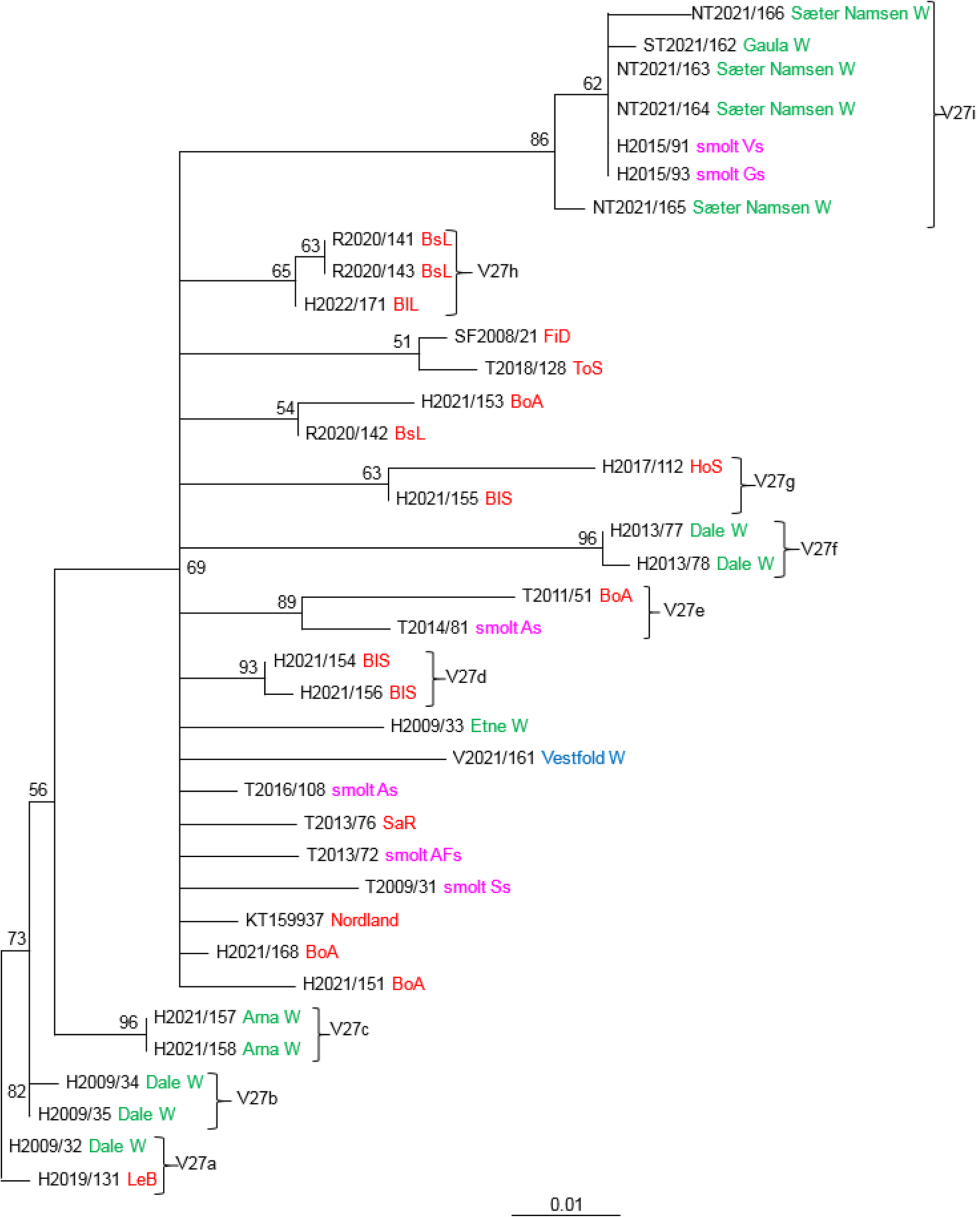
Phylogenetic relationships among SGPV isolates from farmed and wild salmon (N = 37) based on variable region V27*.* A maximum-likelihood quartet puzzling tree is shown. The best-fitting nucleotide substitution model (GTR) was used for maximum-likelihood analysis, and the tree was bootstrapped (50,000 quartet puzzling steps) using TREE_PUZZLE. Branch lengths represent relative phylogenetic distances based on maximum-likelihood estimates. Colored labels indicate the origin of the fish: wild fish in freshwater, green; wild fish in seawater, blue; farmed fish in freshwater, purple; farmed fish in seawater, red. W, wild fish

The phylogeny obtained by analysis of 37 sequences of V27 resulted in nine well-supported clades (Fig. 10). Clades V27d, V27g, and V27h consist only of isolates from farmed salmon, while clades V27b, V27c, and V27f consist of viruses from wild salmon rivers in Hordaland County. V27i includes two viruses from two smolt facilities in Hordaland County (Vs and Gs), and five viruses collected in 2021 from wild salmon in two rivers in Trøndelag. Eight viruses from farmed salmon in the sea, three from farmed smolt, and two from wild salmon (H2009/33 and V2021/161)) have an unresolved position in the phylogeny.

**Fig. 11:**
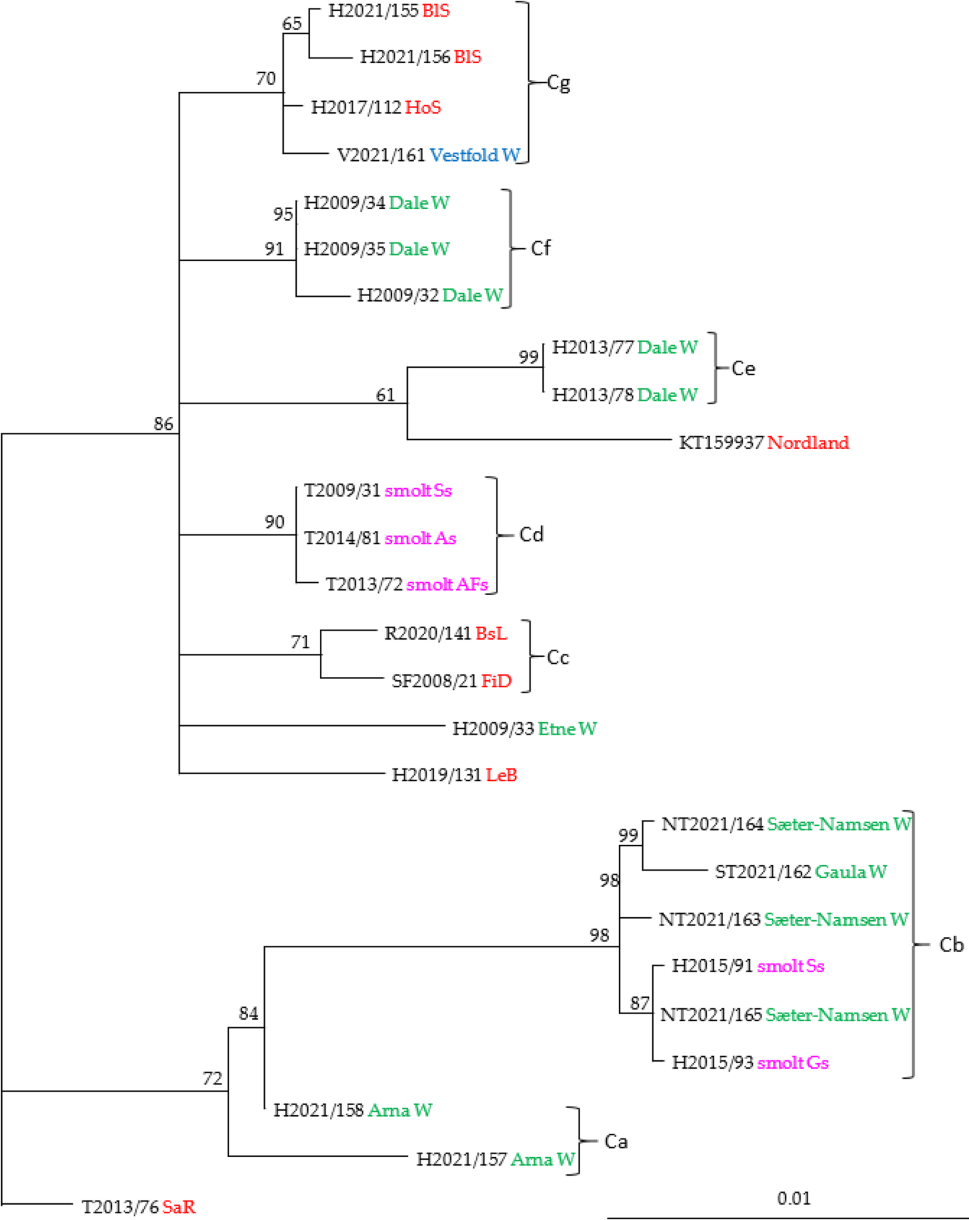
Phylogenetic relationships among SGPV isolates from farmed and wild salmon (N = 26) based on analysis of concatenated sequences from variables V15, V28, V29, and V30 (3453 nucleotides). A maximum-likelihood quartet puzzling tree is shown. The best-fitting nucleotide substitution model (GTR) was used for maximum-likelihood analysis, and the tree was bootstrapped (50,000 quartet puzzling steps) using TREE_PUZZLE. Branch lengths represent relative phylogenetic distances based on maximum-likelihood estimates. Colored labels indicate the origin of the fish: wild fish in freshwater, green; wild fish in seawater, blue; farmed fish in freshwater, purple; farmed fish in seawater, red. W, wild fish

Analysis of a concatenated alignment of V15-V28-V29-V30 including 26 isolates from wild and farmed salmon in seven different counties in Norway resulted in a phylogeny with seven well-supported clades (Fig. 11). Five of the clades (Ca, Cb, Cd, Ce, and Cf) contained only isolates from salmon in freshwater, while clades Cc and Cg contained viruses from salmon collected in the sea. The viruses in Cg were from both farmed and wild salmon. It is important to note that none of the viruses included in this study were from clonal cultures but instead were obtained directly from gill tissues.

## Discussion

### The SGPV genome

The genome of H2015/91 (OQ714485; 240,954 bp) differs in length from that of a virus reported in 2015 (KT159937; 241,564 bp). This difference (610 bp) is primarily due to areas in the genome with different numbers of tandem repeats. All of the gaps in the genome sequence of H2015/91 were filled by Sanger sequencing, but it was not possible to obtain the complete genome sequence of KT159937 [10]. However, it is possible that neither of these sequences represents the complete genome of SGPV due to uncertainties related to tandem repeat sequences and the terminal parts of the genomes. Both of the SGPV genome sequences are much shorter than that of carp edema virus (CEV) (456,821 bp) from koi carp (*Cyprinus carpio*), which is the closest known relative of SGPV [23].

Poxviruses are double-stranded DNA viruses, and while many species have a wide host range, others may be restricted to a single host. It has been suggested that the variability in host range among poxviruses could be linked to presence or absence of “host range genes” [40]. We observed a slight difference in the number of ORFs in H2015/91 and KT159937, with 213 and 210 putative protein-coding genes, respectively.

Knowledge about genes that are important for host specificity (reservoir species), transmission routes, and possible variation in virulence is central for development of strategies to control dissemination of SGPV. Replication of poxviruses in a specific host seems to depend on effective manipulation of the host antiviral response [41]. Between 40 and 50 genes are relatively conserved in all sequenced poxviruses, and an additional 40 genes are present in most chordopoxviruses [42]. Around 75 of these genes have been identified in the SGPV genome based on sequence comparisons [10]. These genes are important for virus transcription, RNA processing, replication, and virion assembly and are located in the central regions of the genome. Genes involved in manipulation of the host immune response and virulence genes are generally located in the terminal regions of the poxvirus genome [1, 24]. The proteins encoded by ORFs in the terminal part of SGPV have yet to be identified. However, a putative metalloendopeptidase of the M60 family has been identified (CDS051). Proteases in this family may act as virulence factors that allow pathogens to break down mucus layers and access host cells [43]. The V28 region included in this study consists of 1329 bp of the reading frame (2718 bp), and in future research, comparisons within the region might provide information about virulence differences among SGPV strains.

### Host specificity

It has already been established that the SGPV is frequently observed on the gills of farmed salmon in both freshwater and seawater [6, 11, 22, 25]. In the present study, we show that, in wild salmon, the highest prevalence is found in salmon in rivers, with a slightly lower prevalence in returning adult salmon in coastal areas. Based on these data, we speculate that the main reservoirs in wild salmon may be found in freshwater (river systems). SGPV was not detected in trout in this study or in other fish species in other studies. The existing knowledge suggests that the SGPV is specific for Atlantic salmon (*Salmo salar*), which should make it easier to identify reservoirs, transmission routes, and virulence markers [22, present study]. SGPV has been found in several European countries and in salmon in eastern North America [25, 44], and the closest known relative to this virus at present is carp edema virus [23]. However, it cannot be excluded that trout (*Salmo trutta*) or other salmonids could be hosts for poxviruses that are closely related to SGPV.

### Genotyping tool for SGPV

A major aim of this study was to identify possible genes or sequence elements that can be used to identify reservoirs and transmission routes for SGPV in Norway. A major focus has been on genes in the terminal regions of the SGPV genome, since these genes might also provide insight into virulence differences. At present, it is difficult to perform repeatable challenge experiments, since it is not possible to culture SGPV from Norwegian salmon [10, 45]. Challenge experiments performed using tissue homogenates have shown that the SGPV isolate used was not lethal unless the fish received an intraperitoneal injection of hydrocortisone [45]. Due to the lack of a reliable system for testing the virulence of different SGPV isolates, the major focus of this study was to locate variations in the viral genome that can be used to identify reservoirs and trace the spread of the virus in Norway.

Molecular tools are of major importance for studies of reservoirs and transmission routes of viruses, and a single multi-locus variable-number tandem-repeat (VNTR) analysis targeting eight loci has been developed for SGPV [25]. However, VNTR analysis cannot be used to identify phylogenetic relationships between strains of a virus. Another problem with using VNTR analysis and capillary electrophoresis is the fact that the length of the PCR products could be determined by different sequences being repeated. One example is the VNTR SGPV-67 [25], in which the repeats found in two different strains of SGPV are of the same length but differ in their nucleotide sequences (ESM_2). Another problem is the fact that it has not been possible to culture SGPV from Norway, and therefore, nothing is known about the stability of the VNTRs in use. In the present study, we tested eight variable regions. These include both intergenic sequences between ORFs (V15, V26, and V27) and sequences containing variation within ORFs (V5, V16, V28, V29, and V30). It is relatively easy to obtain sequences of these regions from gills when the Ct values obtained using the MCP assay are below 27, and they show a significant difference in their relative variability, where V16 seems to be the most variable but provides little information about the relationships between viruses. Phylogenetic analysis based on V28, V29, V30, and V5 resulted in several well-supported clades. Concatenation of sequences obtained directly from gill tissues may introduce errors in phylogenetic analysis if the host is infected simultaneously with two or more strains of SGPV. Hence, before an SGPV isolate is cultured and cloned, its identification should probably be based on separate analysis of the variable regions.

### Geographical reservoirs

Analysis of the eight variable regions showed a wide distribution of closely related SGPV variants from both farmed and wild salmon. However, the viruses obtained from smolt production sites were to a large extent identical within the same geographical area and within smolt farms over a three-year period. The presence of the same virus at the same site over a three-year period could be explained by the presence of “in-house strains” or by an introduction from an unidentified local reservoir, as suggested by Gulla et al. [25]. A close relationship was also observed among viruses collected from salmon in the same year in the rivers Dale, Vosso, Arna, Sæter-Namsen, and Skjern. However, viruses collected from the Dale River in 2009 and 2013 were distinctly different and grouped in separate clades. SGPV from two neighboring rivers, Dale and Vosso, collected in 2009 and 2012, respectively, were also identical (V29c), while another virus from the Vosso River, collected in 2018, grouped in another clade (V29b). The present data suggest that local geographical strains of SGPV may be present in rivers and at smolt production sites, but introduction of new variants into rivers may occur, possibly in connection with salmon returning to spawn.

SGPV isolates obtained from marine farming sites showed no clear indication of their geographical origin and did not provide evidence of the existence of specific geographical strains. SGPV from wild salmon in Vestfold and from marine farms in Rogaland and Hordaland were identical in the analysis of V29 (V29f), and viruses from wild and farmed salmon from the sea from five different counties grouped together in the analysis of V28 (V28b). In the analysis of V15, a total of 18 isolates from 10 locations in freshwater and seawater in three counties (Rogaland, Hordaland, and Troms) were identical (group V15b). The different variants of SGPV in the marine environment seem to have a wide geographical distribution. The lack of geographically distinct SGPV strains in the marine environment could be due to salmon farming and movement of positive smolt between counties in Norway. However, there are also indications of horizontal transmission in the sea, as can be seen in the analysis of V16, where four distinct viruses were obtained from salmon collected from one farm (BoS) in Hordaland. Smolt from the same production site usually have identical or closely related SGPV strains, and this farm had received smolt from only two different smolt production sites. Despite the indications of distinct SGPV strains in reservoirs in fresh water and a lack of distinct geographical strains in the sea, more sequence data are needed before any definite conclusions can be drawn. Nevertheless, the variable regions used in this study are relatively easy to sequence and seem to provide promising information about reservoirs and transmission of SGPV.

### Future research on virulence and severity of disease

Considering that salmon, which live in small, fragmented populations in the wild, seem to be the only hosts for SGPV, it is assumed that SGPV should naturally have low virulence for the host. However, salmon farming has changed the situation, providing a nearly endless availability of new susceptible hosts at high densities in coastal waters. This could lead to a change in virulence, as has been observed for other fish viruses such as isavirus (ISAV), piscine orthoreovirus (PRV1), and Piscine novirhabdovirus (VHSV) [46–54]. To monitor if such changes also occur in SGPV, it is necessary to identify virulence markers in the genome, and this work should focus on the terminal parts of the SGPV genome. V28, part of CDS051, which encodes a putative metalloendopeptidase, could possibly provide such information.

Changes in the genetics of SGPV over time may also give an indication of the impact of salmon farming on the evolution of SGPV. Looking at the information obtained in the present study, it can be concluded that there is a certain degree of stability in variable sequences at freshwater locations. This can be seen in SGPV from smolt production sites containing farmed salmon and rivers with wild salmon. SGPV from two freshwater smolt sites in Troms County showed little or no change over a period of three years based on analysis of the eight variable regions. Identical viruses were also seen in the Sæter-Namsen River in 2021 (Fig. 4). However, comparison of SGPV sequences from the marine environment suggested transmission between regions in Norway, making it possible for recombination between viruses to occur. The presence of large numbers of farmed salmon also increases the size of the virus reservoir and may lead to the evolution of new strains with increased virulence.

### Supplementary Information

Below is the link to the electronic supplementary material.

ESM_1 GenBank accession numbers of the sequences presented in this study (DOCX 54 KB)

ESM_2 Tandem repeats with different nucleotide compositions (SGPV-67, Gullla et al. 2020) (DOCX 959 KB)

Supplementary file3 (AA 325 KB)

## Data Availability

All data are available as part of this publication and in the Supplementary Materials (ESM_1).
