# Supplementary material for: Genotyping tool for salmonid gill pox virus (SGPV) obtained from farmed and wild Atlantic salmon (Salmo salar)
Source: Arch Virol. 2023 Sep 8;168(10):249. doi: 10.1007/s00705-023-05866-8 (PMC10491535; doi:10.1007/s00705-023-05866-8)
Supplement: Supplementary file 1 — ESM_1 GenBank accession numbers of the sequences presented in this study (DOCX 54 KB) [file 705_2023_5866_MOESM1_ESM.docx]

Archives of Virology

Genotyping tool for salmonid gill pox virus (SGPV) obtained from farmed and wild Atlantic salmon (*Salmo salar*).

Are Nylund ^1^, Thomas Kloster-Jensen ^1^, Faezeh Mohammadi ^1^, Erwan Lagadec^1*^ and Heidrun Nylund ^1^

Fish Diseases Research Group, Department of Biological Sciences, University of Bergen, Norway

*****Correspondence: erwan.lagadec@uib.no

| **Code** | **Location** | **Date** | **FW/SW^a^** | **W/F^b^** | **V5** | **V15** | **V16** | **V26** | **V27** | **V28** | **V29** | **V30** |
| --- | --- | --- | --- | --- | --- | --- | --- | --- | --- | --- | --- | --- |
| **2006** |  |  |  |  |  |  |  |  |  |  |  |  |
| SF2006/02 | FiS | Jun | SW | F |  |  | MT904045 |  |  |  |  |  |
| H2006/03 | BsB | Jul | SW | F |  | MT920219 | MT904046 |  |  |  |  |  |
| H2006/04 | BsB | Dec | SW | F |  |  | MT904047 | OP713814 |  |  |  |  |
| H2006/05 | BsB | Dec | SW | F |  |  | OP642703 |  |  |  |  |  |
| **2007** |  |  |  |  |  |  |  |  |  |  |  |  |
| R2007/11 | MoR | Nov | SW | F |  | MT920220 |  | OP713815 |  |  |  |  |
| **2008** |  |  |  |  |  |  |  |  |  |  |  |  |
| SF2008/21 | FiD | Nov | SW | F | MT920251 | MT920221 | MT904048 | OP713816 | OP713851 | OP642730 | OP642787 | OP642844 |
| H2008/22 | HoS | May | SW | F | MT920252 | MT920222 | OP642704 |  |  |  |  |  |
| **2009** |  |  |  |  |  |  |  |  |  |  |  |  |
| T2009/31 | Smolt Ss | May | FW | F | MT920253 | MT920223 | MT904049 | OP713817 | OP713852 | OP642731 | OP642788 | OP642845 |
| H2009/32 | Dale | Nov | FW | W | MT920254 | MT920224 | MT904050 | OP713818 | OP713853 | OP642732 | OP642789 | OP642846 |
| H2009/33 | Etne | Nov | FW | W | MT920255 | MT920225 | MT904051 | OP713819 | OP713854 | OP642733 | OP642790 | OP642847 |
| H2009/34 | Dale | Nov | FW | W | MT920256 | MT920226 | MT904052 | OP713820 | OP713855 | OP642734 | OP642791 | OP642848 |
| H2009/35 | Dale | Nov | FW | W | MT920257 | OP713813 | MT904053 | OP713821 | OP713856 | OP642735 | OP642792 | OP642849 |
| H2009/36 | Etne | Nov | FW | W | MT920258 |  | MT904054 |  |  |  |  |  |
| H2009/37 | Etne | Nov | FW | W |  |  |  |  |  |  | OP642793 |  |
| H2009/38 | Dale | Nov | FW | W |  |  |  |  |  |  | OP642794 |  |
| H2009/39 | Dale | Nov | FW | W |  |  |  |  |  | OP642736 | OP642795 |  |
| H2009/40 | Dale | Nov | FW | W |  |  |  |  |  | OP642737 | OP642796 |  |
| H2009/300 | Dale | Nov | FW | W |  |  |  |  |  |  | OP642797 |  |
| H2009/301 | Dale | Nov | FW | W |  |  |  |  |  | OP642738 |  |  |
| H2009/302 | Etne | Nov | FW | W |  |  |  |  |  | OP642739 |  |  |
| **2010** |  |  |  |  |  |  |  |  |  |  |  |  |
| SF2010/41 | FiD | Oct | SW | F |  | MT920227 | MT904055 | OP713822 |  |  |  | OP642850 |
| H2010/42 | EwL | Jul | SW | F |  | MT920228 | MT904056 |  |  | OP642740 |  |  |
| DK2010/43 | Skjern | May | FW | W |  | MT920229 | MT904057 |  |  | OP642741 | OP642798 |  |
| DK2010/44 | Skjern | May | FW | W |  |  |  |  |  | OP642742 | OP642799 |  |
| DK2010/45 | Skjern | May | FW | W |  |  |  |  |  | OP642743 | OP642800 |  |
| DK2010/48 | Skjern | May | FW | W |  |  |  |  |  | OP642744 |  |  |
| **2011** |  |  |  |  |  |  |  |  |  |  |  |  |
| T2011/51 | Smolt Ss | Jun | FW | F | MT920259 | MT920230 | MT904058 |  | OP713857 | OP642745 |  |  |
| **2012** |  |  |  |  |  |  |  |  |  |  |  |  |
| N2012/61-F227-L3G | Nordland | Sep | SW | F | KT159937 | KT159937 | KT159937 | KT159937 | KT159937 | KT159937 | KT159937 | KT159937 |
| H2012/63 | Vosso | Dec | FW | W |  |  | MT904060 |  |  | OP642746 | OP642801 |  |
| H2012/64 | Vosso | Dec | FW | W |  |  | MT904061 |  |  |  | OP642802 |  |
| **2013** |  |  |  |  |  |  |  |  |  |  |  |  |
| T2013/71 | SaR | May | SW | F | MT920260 | MT920232 |  |  |  |  |  |  |
| T2013/72 | Smolt AFs | Aug | FW | F | MT920261 | MT920233 | MT904065 | OP713823 | OP713858 | OP642747 | OP642803 | OP642851 |
| H2013/73 | BlH | Nov | SW | F |  |  | MT904062 |  |  |  |  |  |
| T2013/74 | SaR | May | SW | F | MT920262 | MT920234 | MT904066 |  |  |  |  |  |
| NT2013/75 | Stjørdal | Aug | FW | W |  |  | OP642705 | OP713824 |  |  |  |  |
| T2013/76 | SaR | May | SW | F | OP619922 | MT920235 | MT904067 | OP713825 | OP713859 | OP642748 | OP642804 | OP642852 |
| H2013/77 | Dale | Nov | FW | W | MT920263 | MT920231 | MT904063 | OP713826 | OP713860 | OP642749 | OP642805 | OP642853 |
| H2013/78 | Dale | Nov | FW | W | MT920264 | OP713789 | MT904064 | OP713827 | OP713861 | OP642750 | OP642806 | OP642854 |
| **2014** |  |  |  |  |  |  |  |  |  |  |  |  |
| T2014/81 | Smolt As | Jan | SW | F | MT920266 | MT920236 | MT904068 | OP713828 | OP713862 | OP642751 | OP642807 | OP642855 |
| SF2014/82 | FiL | Oct | SW | F | MT920265 |  |  |  |  |  |  |  |
| R2014/83 | Lyse | Oct | FW | W |  |  | OP642706 |  |  |  |  |  |
| SF2014/84 | FiL | Sep | SW | F |  |  |  |  |  | OP642752 |  |  |
| T2015/85 | Smolt As | Jan | FW | F |  |  |  |  |  | OP642753 | OP642808 |  |
| T2014/86 | Smolt As | Jan | FW | F |  |  |  |  |  |  |  |  |
| SF2014/87 | FiL | Sep | SW | F |  |  |  |  |  | OP642754 |  |  |
| SF2014/88 | FiL | Sep | SW | F |  |  |  |  |  | OP642755 |  |  |
| **2015** |  |  |  |  |  |  |  |  |  |  |  |  |
| H2015/91 | Smolt Vs | May | FW | F | MT920267 | MT920237 | MT904069 | OP713829 | OP713863 | OP642756 | OP642809 | OP642856 |
| H2015/92 | Smolt Hs | Jan | FW | F | MT920268 | MT920238 | MT904070 |  |  |  | OP642810 |  |
| H2015/93 | Smolt Gs | May | FW | F | MT920269 | MT920239 | MT904071 | OP713830 | OP713864 | OP642757 | OP642811 | OP642857 |
| H2015/94 | Smolt Gs | May | FW | F | MT920270 | MT920240 |  |  |  |  |  |  |
| SF2015/95 | Aarøy | Jul | FW | W |  |  | MT904072 |  |  |  |  |  |
| SF2015/96 | Gaular | Jul | FW | W |  | OP713790 | MT904073 |  |  | OP642758 |  |  |
| H2015/97 | Smolt Vs | May | FW | F |  |  |  |  |  | OP642759 | OP642812 |  |
| H201598 | Smolt Vs | May | FW | F |  |  |  |  |  | OP642760 | OP642813 |  |
| H2015/99 | Smolt Vs | May | FW | F |  |  |  | OP713831 |  | OP642761 | OP642814 |  |
| H2015/100 | Smolt Vs | May | FW | F |  |  |  |  |  | OP642762 | OP642815 |  |
| **2016** |  |  |  |  |  |  |  |  |  |  |  |  |
| H2016/101 | MoH | Jan | SW | F | MT920271 | MT920241 | MT904075 |  |  |  |  |  |
| MR2016/102 | FbS | Jun | SW | F | MT920272 |  | MT904076 |  |  |  |  |  |
| MR2016/103 | FbS | Jun | SW | F | MT920273 | MT920242 | MT904077 |  |  |  |  |  |
| T2016/104 | Smolt As | Dec | FW | F |  | MT920243 | OP642707 |  |  |  |  |  |
| FM2016/105 | Alta | Aug | FW | W |  |  | MT904074 |  |  |  |  |  |
| T2016/107 | Smolt As | Dec | SW | F |  |  |  |  |  |  | OP642816 |  |
| T2016/108 | Smolt As | Dec | FW | F |  | MT920244 | MT904078 | OP713832 | OP713865 | OP642763 |  |  |
| **2017** |  |  |  |  |  |  |  |  |  |  |  |  |
| H2017/111 | HoS | Dec | SW | F | MT920274 | MT920245 | MT904079 |  |  |  |  |  |
| H2017/112 | HoS | Dec | SW | F | MT920275 | MT920246 | MT904080 | OP713833 | OP713866 | OP642764 | OP642817 | OP642858 |
| H2017/113 | MoH | Oct | SW | F | MT920276 | MT920247 | MT904081 |  |  |  |  |  |
| H2017/114 | BoS | Sep | SW | F |  | MT920248 | MT904082 |  |  |  |  |  |
| H2017/115 | BoS | Sep | SW | F |  |  | MT904083 |  |  |  |  |  |
| H2017/116 | BoS | Nov | SW | F |  |  | MT904084 |  |  |  |  |  |
| H2017/119 | BoS | Aug | SW | F |  | MT920249 | MT904085 |  |  |  | OP642818 |  |
|  |  |  |  |  |  |  |  |  |  |  |  |  |
| **2018** |  |  |  |  |  |  |  |  |  |  |  |  |
| H2018/121 | MoT | Jan | SW | F |  | OP713791 | MT904086 |  |  |  | OP642819 |  |
| NT2018/122 | Kvaløy | Jul | SW | W |  |  | MT904088 |  |  |  |  |  |
| NT2018/123 | Steinkjær | Jun | FW | W |  |  | MT904089 |  |  | OP642765 | OP642820 |  |
| NT201/124 | Steinkjær | Jun | FW | W |  |  |  |  |  |  |  |  |
| H2018/126 | Vosso | Nov | FW | Esc | MT920277 | MT920250 | MT904087 |  |  | OP642766 | OP642821 | OP642859 |
| R2018/127 | Lyse | Jun | FW | W | MT920278 |  |  |  |  |  |  |  |
| T2018/128 | ToS | Sep | SW | F | OP619923 |  |  |  | OP713867 | OP642767 | OP642822 |  |
| T2018/129 | ToS | Sep | SW | F |  |  |  |  |  | OP642768 |  |  |
| T2018/130 | ToS | Sep | SW | F |  |  |  |  |  | OP642769 | OP642823 |  |
| **2019** |  |  |  |  |  |  |  |  |  |  |  |  |
| H2019/131 | LeB | Sep | SW | F | OP619924 | OP713792 | OP642708 | OP713834 | OP713868 | OP642770 | OP642824 | OP642860 |
| H2019/132 | LeB | Sep | SW | F | OP619925 | OP713793 |  |  |  | OP642771 | OP642825 |  |
| H2019/134 | LeB | Sep | SW | F |  | OP713794 |  |  |  | OP642772 | OP642826 |  |
| **2020** |  |  |  |  |  |  |  |  |  |  |  |  |
| R2020/141 | BsL | Sep | SW | F | OP619926 | OP713812 | OP642709 | OP713835 | OP713869 | OP642773 | OP642827 | OP642861 |
| R2020/142 | BsL | Sep | SW | F | OP619927 | OP713795 | OP642710 | OP713836 | OP713870 |  |  |  |
| R2020/143 | BsL | Sep | SW | F | OP619928 | OP713796 | OP642711 | OP713837 | OP713871 |  |  |  |
| H2020/144 | MoN | Mar | SW | F | OP619929 | OP713797 |  |  |  | OP642774 | OP642828 |  |
| **2021** |  |  |  |  |  |  |  |  |  |  |  |  |
| H2021/151 | BoA | Sep | SW | F | OP619930 | OP713798 | OP642712 | OP713838 | OP713872 |  | OP642829 |  |
| H2021/152 | BoA | Sep | SW | F |  | OP713799 |  |  |  |  |  |  |
| H2021/153 | BoA | Sep | SW | F | OP619931 | OP713800 | OP642713 | OP713839 | OP713873 |  |  |  |
| H2021/154 | BlS | Sep | SW | F |  | OP713801 | OP642714 | OP713840 | OP713874 |  |  |  |
| H2021/155 | BlS | Sep | SW | F | OP619941 | OP713802 | OP642715 | OP713841 | OP713875 | OP642775 | OP642830 | OP642862 |
| H2021/156 | BlS | Sep | SW | F | OP619932 | OP713803 | OP642716 | OP713842 | OP713876 | OP642776 | OP642831 | OP642863 |
| H2021/157 | Arna | Oct | FW | W | OP619933 | OP713804 | OP642717 | OP713843 | OP713877 | OP642777 | OP642832 | OP642864 |
| H2021/158 | Arna | Oct | FW | W | OP619934 | OP713805 | OP642718 | OP713844 | OP713878 | OP642778 | OP642833 | OP642865 |
| H2021/168 | BoA | Sep |  |  |  |  |  | OQ714491 | OQ714495 | OQ818308 | OQ714498 |  |
| V2021/159 | Vestfold | Jun | SW | W |  |  | OP642719 |  |  |  | OP642834 |  |
| FM2021/160 | Alta | Aug | FW | W |  |  | OP642720 | OP713845 |  |  |  |  |
| V2021/161 | Vestfold | Jun | SW | W | OP619935 | OP713806 | OP642721 | OP713846 | OP713879 | OP642779 | OP642835 | OP642866 |
| ST2021/162 | Gaula | Aug | FW | W | OP619940 | OP713807 | OP642722 | OP713847 | OP713880 | OP642780 | OP642836 |  |
| NT2021/163 | Sæter-Namsen | Aug | FW | W | OP619936 | OP713808 | OP642723 | OP713848 | OP713881 | OP642781 | OP642837 | OP642867 |
| NT2021/164 | Sæter-Namsen | Aug | FW | W | OP619937 | OP713809 | OP642724 | OP713849 | OP713882 | OP642782 | OP642838 | OP642868 |
| NT2021/165 | Sæter-Namsen | Jul | FW | W | OP619938 | OP713811 | OP642725 | OP713850 | OP713883 | OP642783 | OP642839 | OP642869 |
| NT2021/166 | Sæter-Namsen | Sep | FW | W | OP619939 | OP713810 | OP642726 |  | OP713884 | OP642784 | OP642840 | OP642870 |
| V2021/167 | Vestfold | Jul | SW | W |  |  | OP642727 |  |  | OP642785 | OP642841 |  |
| **2022** |  |  |  |  |  |  |  |  |  |  |  |  |
| H2022/171 | BlL | Aug | SW | F |  | OQ714486 | OP642728 | OQ714492 | OQ714496 | OP642786 | OP642842 |  |
| H2022/172 | BlL | Aug | SW | F |  | OQ714487 | OP642729 | OQ714493 |  |  | OP642843 |  |
| V2022/173 | Vestfold | Jun | SW | W |  | OQ714488 | OQ714489 | OQ714494 |  |  |  |  |
| ST2022/174 | Gaula | Jun | FW | W |  |  | OQ714490 |  |  |  |  |  |
| ST2022/175 | Gaula | Jun | FW | W |  |  |  |  |  |  | OQ714499 |  |
|  |  |  |  |  |  |  |  |  |  |  |  |  |
|  |  |  |  |  |  |  |  |  |  |  |  |  |
|  |  |  |  |  |  |  |  |  |  |  |  |  |

^a^: FW=Fresh water; SW=Salt water

^b^: W=Wild salmon; F=Farmed salmon
