# Supplementary material for: Genotyping tool for salmonid gill pox virus (SGPV) obtained from farmed and wild Atlantic salmon (Salmo salar)
Source: Arch Virol. 2023 Sep 8;168(10):249. doi: 10.1007/s00705-023-05866-8 (PMC10491535; doi:10.1007/s00705-023-05866-8)

Archives of Virology

Genotyping tool for salmonid gill pox virus (SGPV) obtained from farmed and wild Atlantic salmon (*Salmo salar*).

Are Nylund ^1^, Thomas Kloster-Jensen ^1^, Faezeh Mohammadi ^1^, Erwan Lagadec^1*^ and Heidrun Nylund ^1^

Fish Diseases Research Group, Department of Biological Sciences, University of Bergen, Norwa

*****Correspondence: erwan.lagadec@uib.no


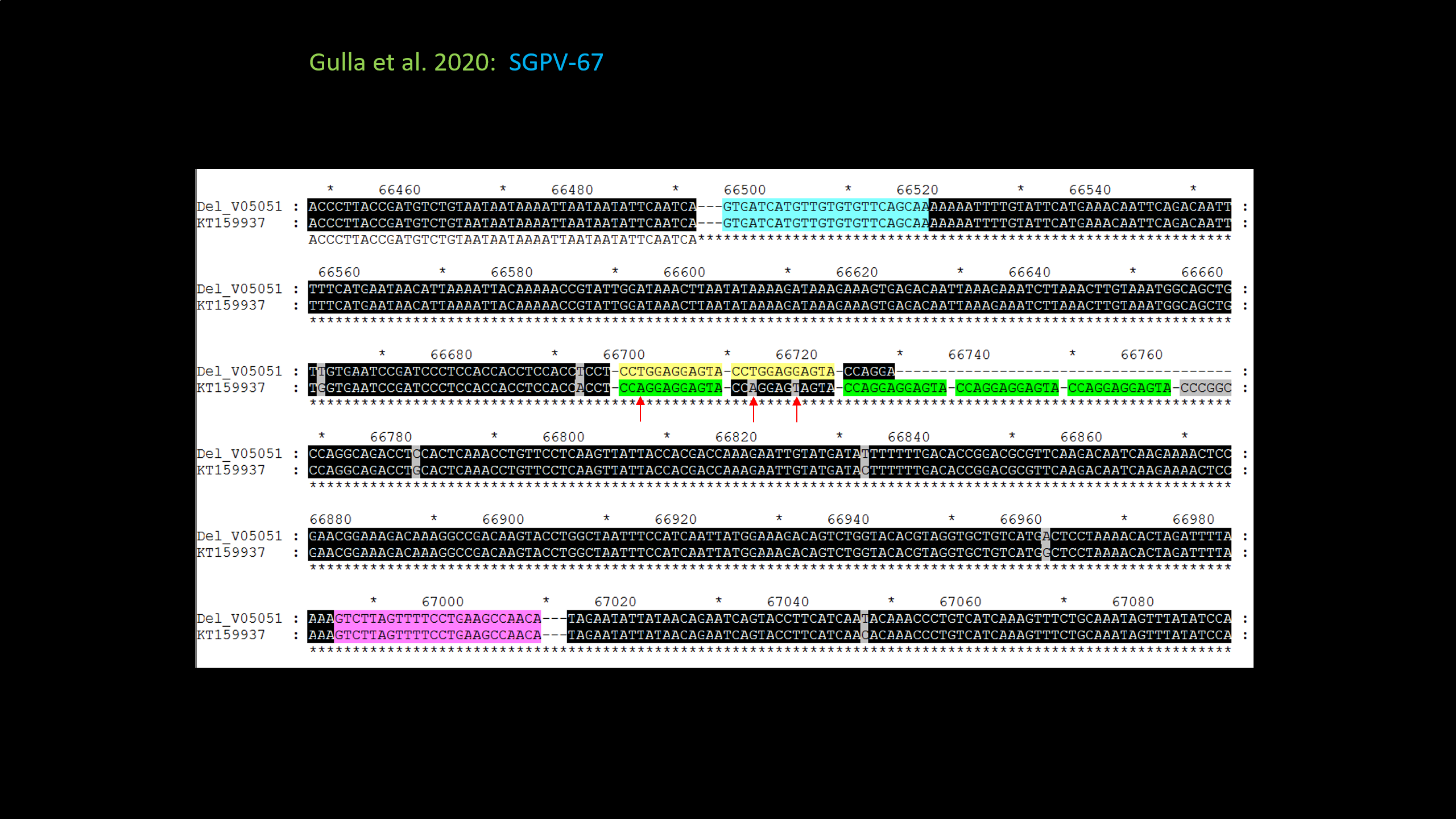

Supplement: Supplementary file 2 — ESM_2 Tandem repeats with different nucleotide compositions (SGPV-67, Gullla et al. 2020) (DOCX 959 KB) [file 705_2023_5866_MOESM2_ESM.docx]
